# Biochemical characterization of Fsa16295Glu from *“Fervidibacter sacchari,”* the first hyperthermophilic GH50 with β-1,3-endoglucanase activity and founding member of the subfamily GH50_3

**DOI:** 10.3389/fmicb.2024.1355444

**Published:** 2024-04-25

**Authors:** Jonathan K. Covington, Nicole Torosian, Allison M. Cook, Marike Palmer, Scott G. Bryan, Nancy O. Nou, Ritesh Mewalal, Miranda Harmon-Smith, Ian K. Blaby, Jan-Fang Cheng, Matthias Hess, Phillip J. Brumm, Nitin K. Singh, Kasthuri Venkateswaran, Brian P. Hedlund

**Affiliations:** ^1^School of Life Sciences, University of Nevada, Las Vegas, NV, United States; ^2^Department of Microbiology, University of Manitoba, Winnipeg, MB, Canada; ^3^US Department of Energy Joint Genome Institute, Lawrence Berkeley National Laboratory, Berkeley, CA, United States; ^4^Department of Animal Science, College of Agricultural and Environmental Sciences, University of California, Davis, Davis, CA, United States; ^5^C5-6 Technologies LLC, Fitchburg, WI, United States; ^6^Biotechnology and Planetary Protection Group, Jet Propulsion Laboratory, California Institute of Technology, Pasadena, CA, United States; ^7^Nevada Institute of Personalized Medicine, University of Nevada, Las Vegas, NV, United States

**Keywords:** hyperthermophile, carboxymethyl curdlan, β-Glucan, β-1,3-endoglucanase, glycoside hydrolase, GH50, CAZyme, *Armatimonadota*

## Abstract

The aerobic hyperthermophile *“Fervidibacter sacchari”* catabolizes diverse polysaccharides and is the only cultivated member of the class *“Fervidibacteria”* within the phylum *Armatimonadota*. It encodes 117 putative glycoside hydrolases (GHs), including two from GH family 50 (GH50). In this study, we expressed, purified, and functionally characterized one of these GH50 enzymes, Fsa16295Glu. We show that Fsa16295Glu is a β-1,3-endoglucanase with optimal activity on carboxymethyl curdlan (CM-curdlan) and only weak agarase activity, despite most GH50 enzymes being described as β-agarases. The purified enzyme has a wide temperature range of 4–95°C (optimal 80°C), making it the first characterized hyperthermophilic representative of GH50. The enzyme is also active at a broad pH range of at least 5.5–11 (optimal 6.5–10). Fsa16295Glu possesses a relatively high *k*_cat_/K_M_ of 1.82 × 10^7^ s^−1^ M^−1^ with CM-curdlan and degrades CM-curdlan nearly completely to sugar monomers, indicating preferential hydrolysis of glucans containing β-1,3 linkages. Finally, a phylogenetic analysis of Fsa16295Glu and all other GH50 enzymes revealed that Fsa16295Glu is distant from other characterized enzymes but phylogenetically related to enzymes from thermophilic archaea that were likely acquired horizontally from *“Fervidibacteria.”* Given its functional and phylogenetic novelty, we propose that Fsa16295Glu represents a new enzyme subfamily, GH50_3.

## 1 Introduction

Thermoactive enzymes are an essential component of hyperthermophiles, which thrive in geothermal environments and have optimal growth at temperatures above 80°C (Adams and Kelly, 2001 Hedlund et al., 2016). These environments, which include marine hydrothermal vents, geysers, fumaroles, and hot springs, host bacteria and archaea that use polysaccharides as carbon and energy sources (Adams and Kelly, 2001 Blumer-Schuette et al., 2008 Strazzulli et al., 2017). A variety of hyperthermophilic bacteria, such as *Thermotoga maritima* and *Caldicellulosiruptor bescii*, can ferment diverse polysaccharides, including lignocellulose, hemicellulose, and glucans, such as β-glucan (Bhalla et al., 2013; Chen et al., 2013; Meng et al., 2016; Crosby et al., 2022).

β-glucans are polysaccharides found in the cell walls of cereal grains and some bacteria and yeast and are composed of glucose residues connected by glycosidic linkages (Wang and Fisher, 2015). The structure and aqueous solubility of β-glucan depend on its origin; for example, β-glucans from plants are branched, relatively soluble, and have alternating β-1,3/1,4 linkages, while those from bacteria are unbranched, insoluble, and have only β-1,3 linkages (Mudgil, 2017; Paudel et al., 2021). Although the specific sources of β-glucans in hot springs have not been investigated, we can better understand how certain hyperthermophilic organisms use β-glucans by studying the enzymes responsible for depolymerizing them.

Glycoside hydrolases (GHs) are a class of enzymes that hydrolyze the glycosidic linkages between sugar residues in carbohydrates, breaking them down into oligosaccharides or even their base monomers (Adams and Kelly, 2001). GHs that degrade β-glucans do so by hydrolyzing either their β-1,3- or β-1,4-glycosidic bonds. These linkages can be cleaved linearly by either exo- or endo-acting enzymes (Santos et al., 2020). The activity of a GH can be predicted to some degree from its amino acid sequence through the presence of conserved active sites and catalytic residues, or by phylogenetic analysis. GHs have been categorized into GH families based on phylogenetic relatedness. These GH families often share one or more identifying features, like conserved catalytic residues, mechanisms, or similar substrates (Henrissat and Bairoch, 1996; Summers et al., 2016). GHs can be classified more broadly into GH clans, comprising GH families that share structural characteristics, and more finely into subfamilies (Henrissat and Bairoch, 1996; Viborg et al., 2019).

One example of a catalytically homogenous GH family is GH family 50 (GH50), of which nearly all known members to date have been classified as β-agarases. These enzymes hydrolyze β-1,4-glycosidic linkages found in agarose, a component of agar alongside agaropectin, to release its degradation products: neoagarohexaose, neoagarotetraose, and neoagarobiose. There are two exceptions to this trend: a β-galactosidase from *Victivallis vadensis* and a β-1,3-glucanase from *Pseudomonas aeruginosa* (Temuujin et al., 2012a; Yi et al., 2018). The earliest studied GH50 was AgaA from *Vibrio* sp. JT0107, which was described as the first endo-β-agarase capable of hydrolyzing both agarose and neoagarotetraose (Sugano et al., 1993). To date, four structures are known from GH50, one forming monomers, two forming homodimers, and one forming homotetramers (Pluvinage et al., 2013; Giles et al., 2017; Zhang et al., 2019; Pluvinage et al., 2020). These structures have C-termini with (β/α)_8_-barrel folds, which are characteristic of the family’s clan, GH clan A (GH-A). Although the catalytic residues of GH50 have not been identified, its designation as a member of GH-A infers the use of two conserved glutamic acid residues as the acid/base catalyst and nucleophile (Kumar et al., 2019). Current research surrounding GH50s has focused on their potential for the production of neoagarobiose, which is used as a skin moisturizer and melanoma whitener (Lee et al., 2008). However, GH50 is an understudied family. Of the 1,631 GH50s that are currently deposited in the carbohydrate-active enzyme (CAZy) database (cazy.org), only 30 have been functionally characterized (Drula et al., 2022). The β-galactosidase (VadG925) and β-1,3-glucanase (PaBglu50A) set a precedent that GH50 may be more catalytically diverse than currently known. The study of GH50s from novel or understudied microbes broadens our understanding of the true diversity of this GH family.

We recently isolated *“Fervidibacter sacchari,”* an aerobic hyperthermophile (T_opt_ 80°C) belonging to the phylum *Armatimonadota* (Nou et al., 2024). *“F. sacchari”* uses at least 16 diverse polysaccharides as sole carbon sources and electron donors, including glucans, hemicelluloses, and plant biomass. The genome of *“F. sacchari”* has 117 genes encoding GH domains across 49 GH families, including two that were annotated as members of GH50, designated Fsa16295Glu and Fsa11540. Since the optimal growth temperature of *“F. sacchari”* exceeds the optimal temperature of the most thermophilic GH50 enzyme characterized to date, AgrA from *Agarivorans* sp. AG17 (T_opt_ 65°C) (Nikapitiya et al., 2010), we probed the temperature optimum and thermostability of Fsa16295Glu and compared its properties to other GH50 enzymes. To do this, we codon-optimized the gene encoding Fsa16295Glu for expression in *Escherichia coli*, synthesized it, ligated it into the pET21b-GB1 vector, and expressed it in *E. coli*. The enzyme was then purified and used to determine its substrates, optimal conditions, kinetic parameters, and biochemical characteristics. Finally, we examined the evolutionary history of Fsa16295Glu within the GH50 family by conducting a comprehensive phylogenetic analysis of GH50 enzymes. We show that Fsa16295Glu is indeed hyperthermophilic (T_opt_ 80°C) and is a β-1,3-endoglucanase with only weak activity on agarose. Because of this unusual activity and its unique phylogenetic position, we propose that Fsa16295Glu represents a novel GH50 subfamily herein designated as GH50_3.

## 2 Materials and methods

### 2.1 Strains, gene synthesis, heterologous expression, enzyme purification, and computed structure models

*“F. sacchari”* inhabits ~80°C sediments in Great Boiling Spring, NV, USA, (Costa et al., 2009 Cole et al., 2013) and has been enriched *in situ* following lignocellulose addition (Peacock et al., 2013). A single strain of *“F. sacchari,”* strain PD1, was isolated following *in situ* and laboratory enrichment by using optical tweezers followed by aerobic growth at 80°C with polysaccharides as the sole carbon source and electron donor, and its whole genome was assembled (Nou et al., 2024) (GCA_030520105.1). Since the name *Fervidibacter sacchari* has not yet been validly published, we use the name in quotations in this manuscript. The amino acid sequence of Fsa16295Glu was downloaded and submitted to the dbCAN2 meta server to determine the GH family of Fsa16295Glu (Zhang et al., 2018). The amino acid sequence was then submitted for annotation using SignalP (Teufel et al., 2022) to determine the presence of a signal sequence. A MAFFT-DASH (Katoh et al., 2019) multiple sequence alignment of seven GH50 amino acid sequences (Fsa16295Glu, Fsa11540, Aga50D, AgaA, AgrA, PaBglu50A, and VadG925) was generated and visualized in Jalview v2.11.2.6 (Waterhouse et al., 2009) to confirm the presence of two conserved glutamic acid residues. The gene encoding Fsa16295Glu was codon-optimized using an *E. coli* codon frequency table (Oberortner et al., 2017). This refactored sequence was synthesized (Twist BioScience, CA) inclusive of 30mer flanking linkers enabling assembly into the BamHI site of the pET21b vector with NEBuilder Hi-Fi Assembly (Novagen, Madison, WI; New England Biolabs, Ipswich, MA). The final construct, which contained a 6 × His tag, a GB1 solubility tag, and a TEV protease cleavage site at the protein N-terminus, was sequence verified on the Pacific Biosciences Sequel IIe platform and transformed into *E. coli* T7 Express cells (New England Biolabs, Ipswich, MA) for expression.

*E. coli* cells containing the gene encoding Fsa16295Glu were grown on Luria-Bertani (LB) agar plates (10 g tryptone, 5 g yeast extract, 5 g NaCl per L) containing 100 μg/mL ampicillin. For expression, LB broth with 100 μg/mL ampicillin was inoculated and incubated at 37°C with shaking at 225 rpm until reaching an OD_600_ of 0.6–0.8. Isopropyl-β-thiogalactoside (IPTG) was added to a concentration of 0.5 mM and the culture was incubated overnight at 37°C with shaking at 150 rpm. The cells were centrifuged at 16,100 × *g*, resuspended in lysis buffer (50 mM Tris–HCl, 100 mM NaCl, pH 7.0), and lysed by sonication for 40 s with 40% cycle and level 4 output using a Branson 450 Sonifier (Branson Ultrasonics, Brookfield, CT, United States). The crude lysate was clarified by centrifugation at 16,100 × *g* to isolate the soluble fraction.

Fsa16295Glu was purified by heating the soluble fraction to 80°C in a heat block for 30 min, followed by centrifugation at 16,100 × *g* to remove denatured host proteins in the pellet. Fsa16295Glu expression and purity were assessed using 7.5% sodium dodecyl sulfate-polyacrylamide gel electrophoresis (SDS-PAGE). The molecular weight was estimated using a BriteRuler Pre-stained Protein Ladder (Abcam, Cambridge, United Kingdom). The ability of Fsa16295Glu to oligomerize was assessed using 7.5% native PAGE alongside a PageRuler™ Plus Pre-stained Protein Ladder (Thermo Scientific, Vilnius, Lithuania). Protein concentration was assessed by a Qubit® Protein and Protein Broad Range Assay Kit (Thermo Fisher Scientific, Waltham, MA, USA) using a Qubit® 3.0 Fluorometer (Thermo Fisher Scientific, Waltham, MA, USA).

Computed structure models (CSMs) of Fsa16295Glu were generated using AlphaFold 2 via the ColabFold platform integrated with ChimeraX v 1.5 using default settings (Pettersen et al., 2021; Mirdita et al., 2022). A total of five Fsa16295Glu CSMs were generated: (i) native and mature Fsa16295Glu (signal sequence removed); (ii) non-native Fsa16295Glu containing an N-terminal 6 × His tag, GB1 solubility tag, and a TEV protease site; (iii) homodimeric native, and mature Fsa16295Glu; (iv) homotrimeric native, and mature Fsa16295Glu; and (v) homotetrameric native, and mature Fsa16295Glu. The resultant structures were visualized and edited in ChimeraX v 1.5, and the predicted aligned error (PAE) plots of the multimeric CSMs were analyzed to gain insight into the potential of Fsa16295Glu to homooligomerize.

### 2.2 Enzyme activity assays

To determine the enzyme activity of Fsa16295Glu, the following potential substrates were tested initially: chondroitin sulfate (Alfa Aesar), colloidal chitin as prepared according to Hsu and Lockwood (1975) (Beantown Chemicals), brown algae fucoidan (BestVite), lupin galactan (Megazyme), gellan gum (Serva), karaya gum (Sigma), locust bean gum (Spectrum), xanthan gum (Sigma), birch wood xylan (Sigma), tamarind xyloglucan (CarboMer), oat β-glucan (Megazyme), oyster glycogen (TCI), potato starch (J.T. Baker), and ammonia fiber expansion (AFEX)-pretreated corn stover, *Miscanthus*, and sugarcane bagasse (DuPont). These 16 substrates were chosen based on their use by *“F. sacchari”* as sole carbon sources and electron donors. Agarose (VWR Chemicals) was included in the screen to test for β-agarase activity that would be consistent with GH50; carboxymethyl curdlan (CM-curdlan) (Megazyme) was included to test for β-1,3-glucanase activity; laminarin from *Eisenia bicyclis* (TCI America) was included to test for β-1,3/1,6-glucanase activity; and carboxymethyl cellulose (CMC) (Spectrum) was included to test for β-1,4-glucanase activity. The substrates (0.5% w/v) and enzyme (141 μg/mL) or empty vector control were mixed 1:1 in microplate wells (final volume 40 μL) and then sealed and incubated overnight at 80°C with a beaker of water to prevent evaporation. To measure the quantity (μmol) of reducing sugars released by the reactions, 3,5-dinitrosalicylic acid (DNS) solution (0.25 g DNS, 75 g sodium potassium tartrate, 50 mL 2 M sodium hydroxide solution, brought to 250 mL using ultrapure water) was added 4:1 to each reaction mixture (final volume 200 μL). The plate was wrapped in foil and incubated at 100°C for 20 min (Kim et al., 2010). Absorbance was measured using a SpectraMax Plus Plate Reader at 570 nm. A second screen with robust quantitation was conducted with Fsa16295Glu (141 μg/mL) in triplicate and compared with the empty vector control using an unpaired t-test. Additionally, a parallel screen was conducted using Fsa16295Glu pretreated with TEV protease (New England Biolabs, Ipswich, MA) on chondroitin sulfate (Alfa Aesar), colloidal chitin (Beantown Chemicals), lupin galactan (Megazyme), gellan gum (Serva), karaya gum (Sigma), locust bean gum (Spectrum), xanthan gum (Sigma), birch wood xylan (Sigma), beech wood xylan (Megazyme), tamarind xyloglucan (CarboMer), oat β-glucan (Megazyme), and potato starch (J.T. Baker). A DNS standard curve was generated using glucose at 0, 5, 10, 15, 20, and 25 mM.

To determine how much Fsa16295Glu depolymerized its substrates, acid hydrolysates of CM-curdlan, oat β-glucan, laminarin, and agarose were prepared by heating 1% solutions of each substrate in 2 M sulfuric acid at 121°C for 60 min followed by neutralization with 5 M sodium hydroxide. The acid hydrolysates were incubated with water instead of Fsa16295Glu, and the amount of reducing sugars released from exhaustive overnight degradation by Fsa16295Glu was compared with the reducing sugar content of the acid hydrolysates normalized to enzyme assay concentrations.

The β-glucosidase activity of Fsa16295Glu was assessed using the *para*-nitrophenyl (*p*NP)-linked substrate *p*NP-β-D-glucopyranoside (*p*NPG) (Megazyme). *p*NPG (20 μg/mL) was mixed with Fsa16295Glu (141 μg/mL) and incubated for 30 min at 80°C, and the reaction was terminated with 2% w/v disodium phosphate solution (pH 12.0). Bond cleavage was assessed as an increase in absorbance at 400 nm, indicating substrate hydrolysis. A standard curve was generated using a range of *para-*nitrophenol concentrations (0, 6, 12, 18, 24, and 30 μg/mL). In addition to laminarin, β-1,3/1,6-glucan cleavage by Fsa16295Glu was assessed using the Enzymatic Yeast Beta-Glucan test kit (Megazyme) with active dry yeast (Ward’s Science) by substituting Fsa16295Glu for Glucamix.

### 2.3 Biochemical characterization and kinetic parameters

To determine the temperature range and optimum of Fsa16295Glu, the enzyme was incubated with 0.5% w/v oat β-glucan at a range of temperatures (4, 20, 30, 50, 60, 70, 80, 90, and 95°C). The resulting solution was analyzed as described before using a DNS solution. To determine the thermostability of the enzyme, Fsa16295Glu was incubated for 1 h at either 70, 80, 90, or 100°C, then incubated at 80°C with oat β-glucan and tested using the DNS assay. To determine the pH range and optimum of Fsa16295Glu, the enzyme was expressed and purified in 50 mM buffers at different pHs in 0.5 intervals: 2-morpholin-4-ylethanesulfonic acid (MES) (5.5–6.5), 2-amino-2-(hydroxymethyl)propane-1,3-diol (Tris) (7.0–9.0), 2-(cyclohexylamino)ethane-1-sulfonic acid (CHES) (9.5–10.0), and 3-(cyclohexylamino)propane-1-sulfonic acid (CAPS) (10.5–11.0), all with 100 mM NaCl. Oat β-glucan was dissolved in the same buffers to capture the whole pH range. Assays were conducted with 0.5% w/v oat β-glucan in triplicate and then analyzed as previously described using the DNS method. The temperature and pH ranges and optima and thermostability were determined using one-way analysis of variance (ANOVA) with *post-hoc* Tukey’s honestly significant difference (HSD) tests (*p* ≤ 0.05).

To determine the Michaelis–Menten kinetic parameters *k*_cat_, and *k*_cat_/K_M_ of Fsa16295Glu, CM-curdlan or oat β-glucan was mixed with purified Fsa16295Glu (141 μg/mL) at a range of concentrations (156, 313, 625, 1,250, 2,500, and 5,000 μg/mL) and incubated for 30 min. Reducing sugars released by the reactions were quantified using the DNS assay as before. Lineweaver–Burk plots were generated from these data to calculate V_max_, K_M_, *k*_cat_, and *k*_cat_/K_M_ (Ochs, 2022).

### 2.4 Phylogenetic analysis of GH50

To perform a phylogenetic analysis of all GH50s, all sequences belonging to GH50 in the CAZy database (Drula et al., 2022) were extracted from the National Center for Biotechnology Information (NCBI; https://www.ncbi.nlm.nih.gov) using the Batch Entrez site (https://www.ncbi.nlm.nih.gov/sites/batchentrez). BioEdit v. 7.2.5 (Hall, 1999) was used for visualizing and editing all data matrices. After the removal of all duplicate sequences, protein sequences of less than 50 amino acids were also removed. Fsa16295Glu along with all other GH50 homologs from the class *Fervidibacteria* (133 in total) was added to the dataset, resulting in 1,638 protein sequences. A multiple sequence alignment was generated using the MAFFT online server v. 7 (Katoh et al., 2019) with the MAFFT FFT-NS-2 progressive alignment algorithm (Rozewicki et al., 2019). A smaller alignment was similarly generated to more easily visualize the relationship between Fsa16295Glu and its closest relatives. For this smaller phylogeny, the MAFFT-DASH structural alignment algorithm (Katoh et al., 2019) was used instead to bring structural information into account. The larger alignment was then subjected to an approximate maximum likelihood analysis with FastTree v. 2.1.11 (Price et al., 2010) using Shimodaira–Hasegawa approximate likelihood ratio test (SH-aLRT) branch support values (Guindon et al., 2010) from 1,000 replicates. The smaller dataset was subjected to a true maximum likelihood analysis with IQTree v. 2.2.0 (Minh et al., 2020) using SH-aLRT branch support values from 1,000 replicates and ultrafast bootstrapping (UFBoot) branch support from 1,000 replicates. The obtained phylogenies were visualized using the Interactive Tree of Life v. 6.7 website (https://itol.embl.de) and edited in Inkscape v. 0.92.4 (https://www.inkscape.org).

## 3 Results

### 3.1 Computed structure modeling, heterologous expression, and substrate screening

**Figure 1:**
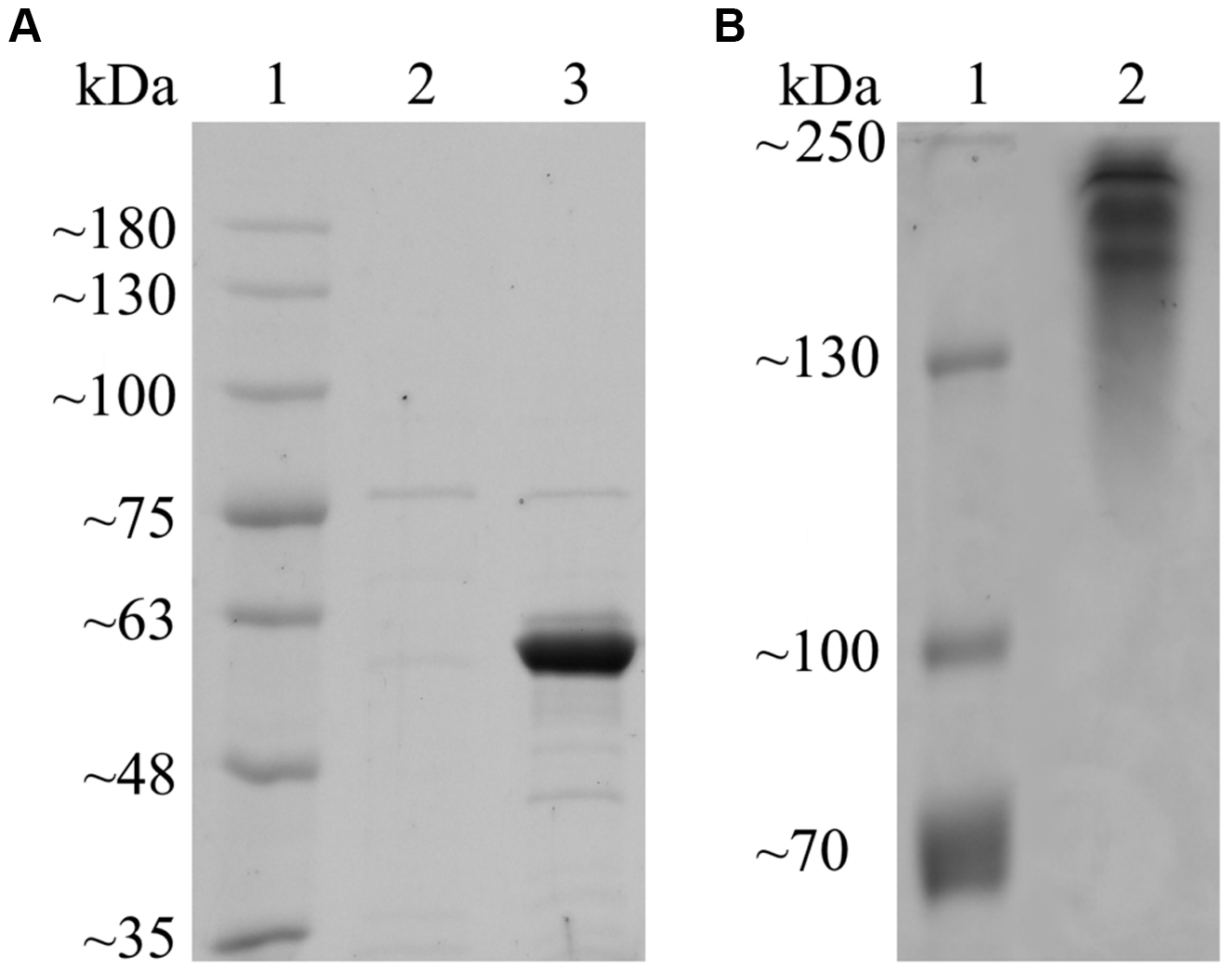
SDS-PAGE and native PAGE of purified Fsa16295Glu. **(A)** Proteins were resolved on a 7.5% polyacrylamide SDS-PAGE gel. Lane 1: BriteRuler Pre-stained Protein Ladder (Abcam, Cambridge, United Kingdom); 2: lysate from empty vector control following heat-purification procedure; 3: heat-purified non-native Fsa16295Glu containing N-terminal 6 × His tag, GB1 solubility tag, and TEV protease site. **(B)** Proteins were resolved on a 7.5% polyacrylamide native PAGE gel. Lane 1: PageRuler™ Plus Pre-stained Protein Ladder (Thermo Scientific, Vilnius, Lithuania); 2: heat-purified non-native Fsa16295Glu containing N-terminal 6 × His tag, GB1 solubility tag, and TEV protease site. Both gels were subsequently stained using Coomassie brilliant blue.

Following annotation by HMMER (E-value = 3.90 × 10^−102^; cutoff = 1 × 10^−15^) and dbCAN-sub (E-value = 5.00 × 10^−134^; cutoff = 1 × 10^−15^), Fsa16295Glu was determined to belong to GH50 with no carbohydrate-binding modules or other annotated domains. Fsa16295Glu had an N-terminal signal sequence (MSWTRREFVKLVGFATTFAGASCRSEG) that was predicted using SignalP to belong to the twin-arginine translocation (TAT) pathway, suggesting that Fsa16295Glu is secreted to the extracellular space as a folded protein (Ren et al., 2016). A multiple sequence alignment of seven GH50 proteins (Fsa16295Glu, Fsa11540, Aga50D, AgaA, AgrA, PaBglu50A, and VadG925) revealed that Fsa16295Glu has two residues, Glu192 and Glu354, that align with known catalytic residues of other clan GH-A enzymes and are highly conserved among the clan (Kumar et al., 2019). Similarly, Fsa11540 has the conserved residues at Glu409 and Glu575 (Supplementary Figure S1). After expression and heat purification, the purity of Fsa16295Glu was estimated by SDS-PAGE to be >90%, and the molecular weight was estimated at 60 kDa (Figure 1). The size of the recombinant protein was affected by the removal of the TAT signal sequence (3 kDa) and the addition of a GB1 solubility tag (6 kDa), a 6 × His tag (1 kDa), and a TEV protease site (1 kDa). These modifications made the recombinant Fsa16295Glu slightly larger than the predicted native size of 52 kDa (Supplementary Figure S2). The resolved native PAGE gel revealed three bands that are apparently larger than 130 kDa, suggesting the formation of multimeric structures (Figure 1).

The CSM of native, mature Fsa16295Glu had low predicted aligned error (PAE) scores (0–5) indicative of high confidence in the accuracy of the CSM (Supplementary Figures S3A,B). Glu192 and Glu354 (Glu165 and Glu327 in the mature form) are found in a tunnel through the structure’s center, marking this as the candidate active site (Supplementary Figure S3C). Furthermore, the residues surrounding the active site tunnel had higher PAE scores (2.5–5), suggesting greater structural flexibility, supporting this tunnel as the active site. The CSM of non-native Fsa16295Glu containing an N-terminal 6 × His tag, GB1 solubility tag, and a TEV protease site (Supplementary Figure S3D) also had low PAE scores (0–5) in the catalytic domain, indicative of normal predicted folding, and was the form of Fsa16295Glu used for biochemical characterization. CSMs of the homodimeric and homotrimeric forms of Fsa16295Glu predicted asymmetric multimers (Supplementary Figure S3E), supporting multimerization of Fsa16295Glu observed by native PAGE. However, the PAE plot of the homodimer had low PAE scores within each monomer (0–5), and low-to-moderate PAE scores between monomers (5–15), while the PAE plots of the homotrimer and homotetramer showed high PAE scores between monomers (25–30). These results suggest low confidence in a homotrimer or homotetramer structure and moderate confidence in a homodimer structure of Fsa16295Glu (Supplementary Figure S3F). The CSM of Fsa16295Glu was similar to the CSMs of three uncharacterized β-agarases in the AlphaFold Protein Structure Database (Jumper et al., 2021; Varadi et al., 2022), all from the bacterial phylum *Armatimonadota*. These three predicted enzymes also had high Protein Basic Local Alignment Search Tool [NCBI BLASTp] scores with Fsa16295Glu: A0A7C3N829 [UniProt accession number], (E-value = 0, percent identity = 92.68%; A0A7C2VJI7, E-value = 0, percent identity = 78.17%; and A0A2H5XB18, E-value = 0, percent identity = 77.90%) (Kato et al., 2018; Zhou et al., 2020). Previously experimentally validated GH50 structures most similar to Fsa16295Glu were Aga50D, a homotetrameric β-agarase from *Saccharophagus degradans* 2–40 (E-value = 6 × 10^−54^, percent identity = 33.42%) (Kim et al., 2010; Pluvinage et al., 2013) and AgWH50C, a homodimeric β-agarase from *Agarivorans gilvus* WH0801 (E-value = 3 × 10^−53^, percent identity = 33.24%) (Liu et al., 2012; Zhang et al., 2019). Aga50D homotetramers possess similar tunnel-shaped active sites on each molecule that contain conserved GH-A catalytic residues Glu695 and Glu534, which align with Glu354 and Glu192 from Fsa16295Glu, respectively. Additionally, Fsa16295Glu possesses other GH50 active site residues present in both Aga50D and AgWH50C: Glu417, Arg413, Asn191, and Phe403 in Fsa16295Glu; Glu757, Arg752, Asn533, and Phe742 in Aga50D; Glu705, Arg700, Asn483, and Phe690 in AgWH50C. Unlike Aga50D and AgWH50C, which have conserved N-terminal carbohydrate-binding module (CBM) domains, Fsa16295Glu does not have an annotated N-terminal CBM domain. Overall, these shared active site residues provide additional support for the putative active site tunnel of Fsa16295Glu. The catalytic mechanisms of Aga50D and AgWH50C were described as retaining and putatively retaining, respectively, based on the role of the conserved Aga50D active site glutamic acid residues as a nucleophile (Glu695) and proton donor (Glu534), which is consistent with the retaining mechanism of GH-A enzymes (Kumar et al., 2019). Thus, we putatively identified the catalytic mechanism of Fsa16295Glu to be retaining, wherein Glu354 acts as a nucleophile and Glu192 serves as a proton donor.

**Figure 2:**
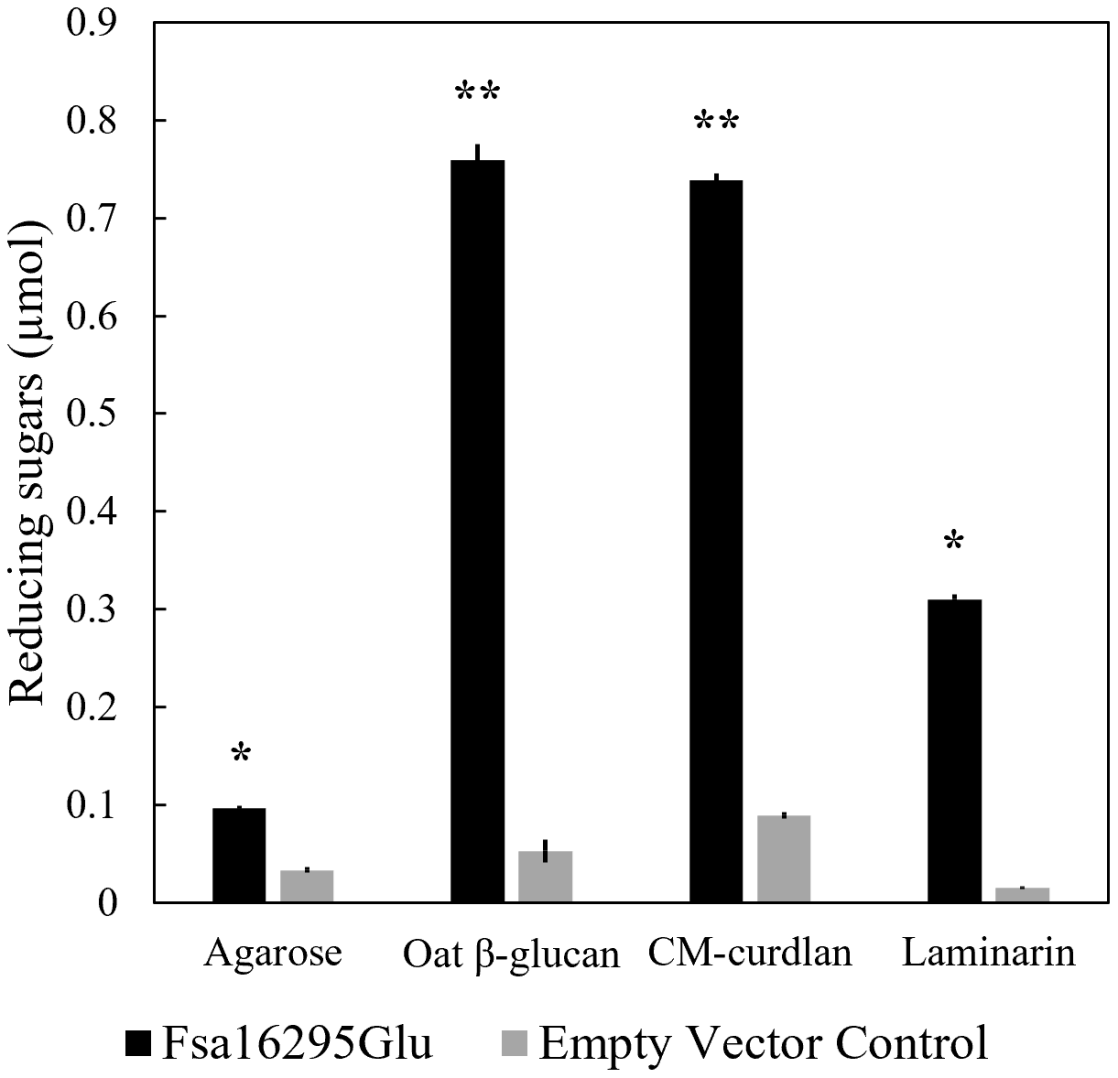
Substrate specificity. Fsa16295Glu is active on agarose, β-glucan, CM-curdlan, and laminarin compared with an empty vector control after a 20-h incubation (* *p* ≤ 0.00005 and ** *p* ≤ 0.000005 via an unpaired *t*-test). Error bars are based on standard deviation.

After screening with 20 potential substrates using the DNS method, Fsa16295Glu was highly active on CM-curdlan and oat β-glucan, and slightly active on laminarin and agarose (Figure 2). Removal of N-terminal 6 × His and GB1 solubility tags via TEV protease site cleavage had no effect on activity with oat β-glucan or any of the 11 other substrates that were tested. Complete acid hydrolysis of CM-curdlan, oat β-glucan, laminarin, and agarose, yielded 0.93 ± 0.01, 1.16 ± 0.08, 0.89 ± 0.01, and 0.66 ± 0.01, μmol of reducing sugars, suggesting that Fsa16295Glu incubated overnight at 80°C hydrolyzed 79.2% of the glycosidic bonds in CM-curdlan, 65.6% of those in oat β-glucan, 35.0% of those in laminarin, and 14.6% of those in agarose. Fsa16295Glu released 0.37 μmol of *p*NP from *p*NPG after 30 min, confirming that it had β-glucosidase activity (Supplementary Figure S4). Fsa16295Glu was not active on yeast β-glucan, CMC, chondroitin sulfate, colloidal chitin, brown algae fucoidan, lupin galactan, gellan gum, karaya gum, locust bean gum, xanthan gum, birch wood xylan, tamarind xyloglucan, oyster glycogen, potato starch, or the AFEX-pretreated substrates corn stover, *Miscanthus*, or sugarcane bagasse. The absence of activity on the insoluble AFEX-pretreated substrates is consistent with Fsa16295Glu not having a CBM domain and suggests that Fsa16295Glu may be involved in oligosaccharide degradation following initial hydrolysis of insoluble polysaccharides by GHs containing CBM domains.

### 3.2 Biochemical characterization and kinetic parameters

**Figure 3:**
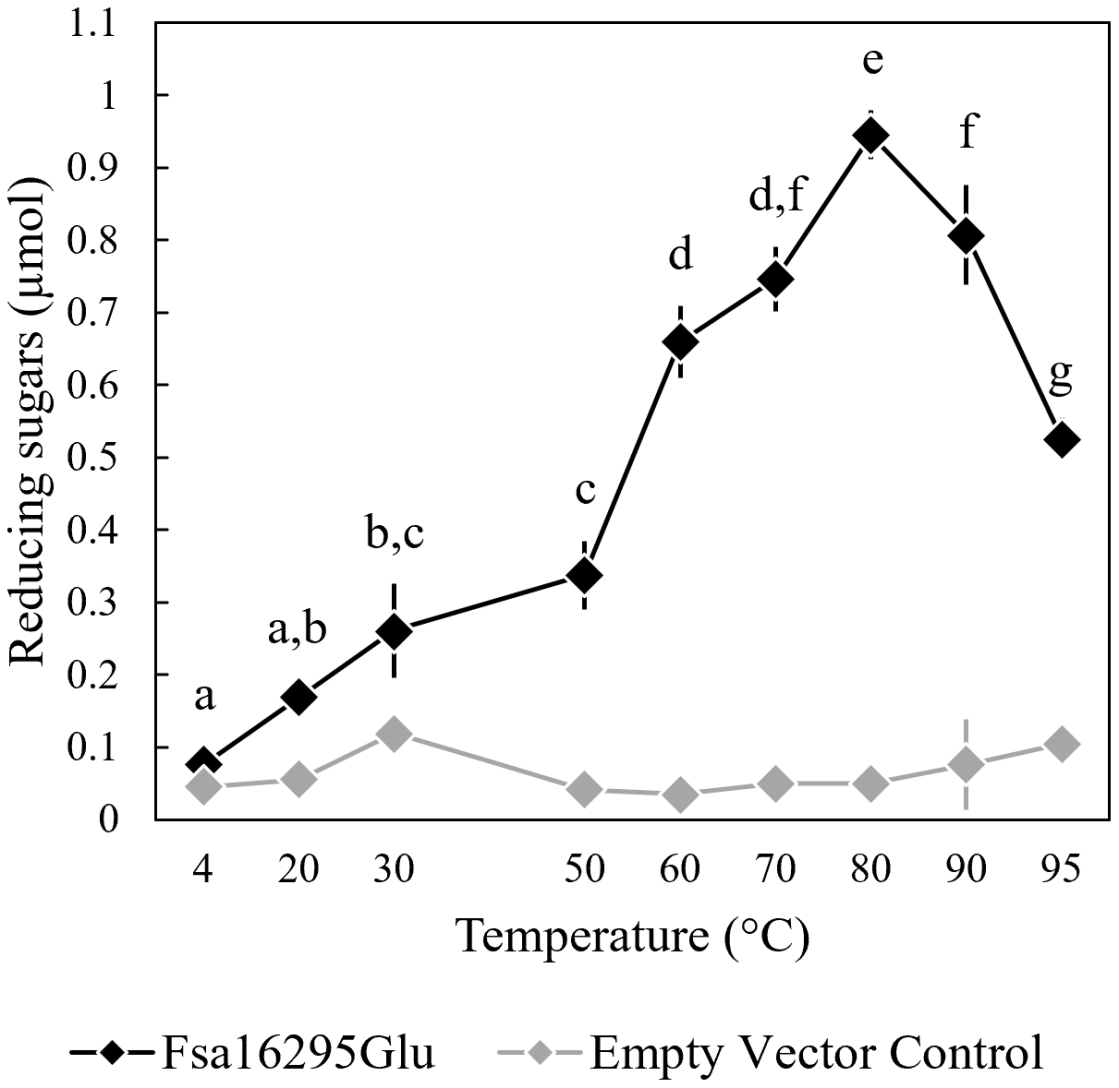
Temperature range and optimum of Fsa16295Glu. Fsa16295Glu was most active on oat β-glucan between 60 and 95°C, and optimally active at 80°C. Temperatures with a shared letter are not significantly different (*p* ≤ 0.05 via a one-way ANOVA with *post-hoc* Tukey’s HSD). Error bars are based on standard deviation. The enzyme was active at the full range of temperatures tested, 4–95°C, based on comparisons to an empty vector control at each temperature (*p* < 0.05 via unpaired *t*-tests).

**Figure 4:**
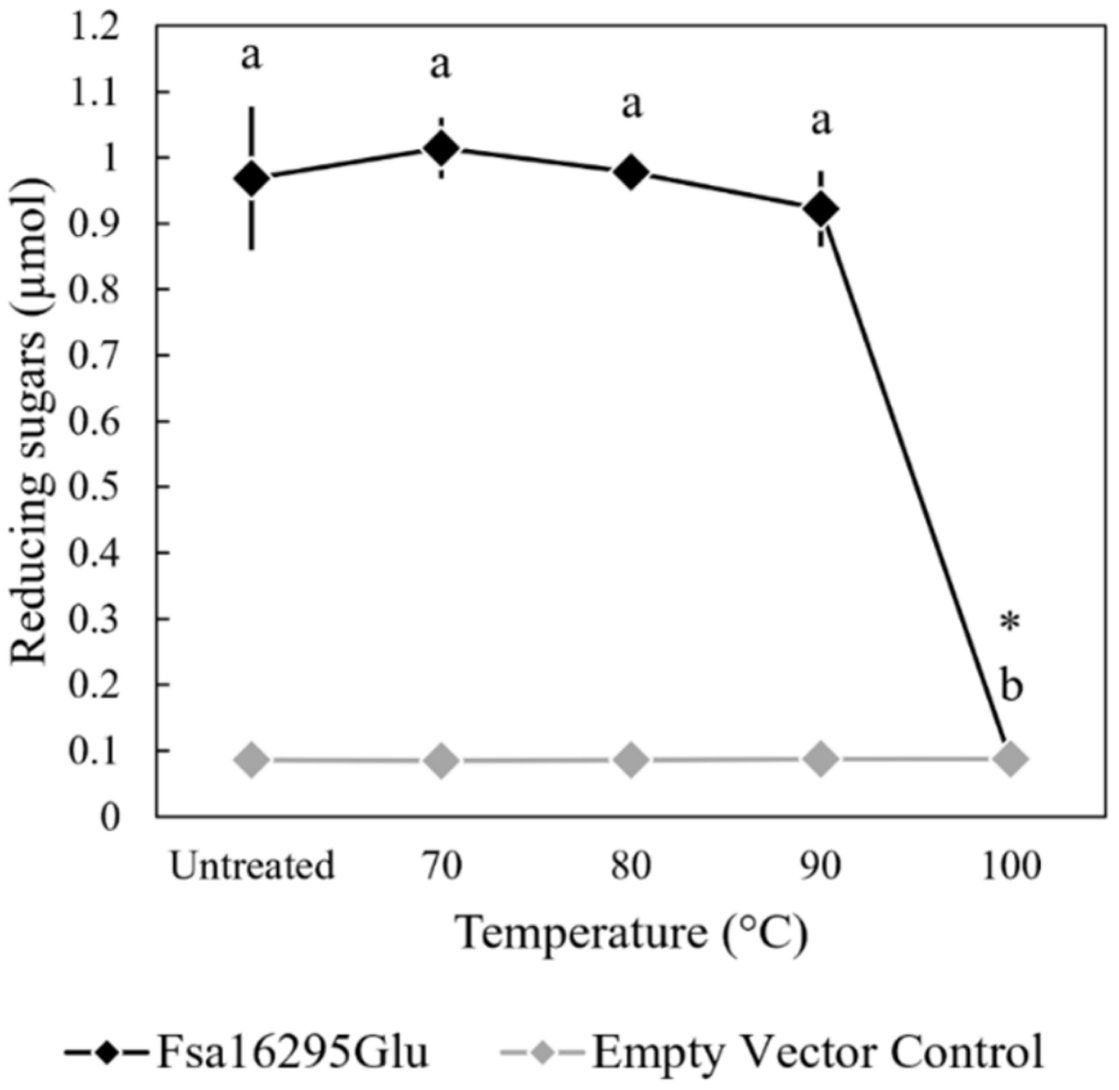
Thermostability of Fsa16295Glu. Fsa16295Glu was stable up to 90°C for 1 h, while incubation at 100°C for 1 h resulted in the complete loss of activity on oat β-glucan. Temperatures with a shared letter are not significantly different (*p* ≤ 0.05 via a one-way ANOVA with *post-hoc* Tukey’s HSD) and Fsa16295Glu treated at 100°C for 1 h was not significantly different from an empty vector control (*p* > 0.05 via an unpaired *t*-test). Error bars are based on standard deviation.

When tested with oat β-glucan at a range of temperatures and pH values, Fsa16295Glu was shown to be hyperthermophilic with a broad pH range. Fsa16295Glu was active between 4 and 95°C with an optimum of ~80°C (Figure 3). Fsa16295Glu was thermostable up to at least 90°C, with similar amounts of reducing sugars released following a 1-h treatment at 70°C (1.02 μmol), 80°C (0.98 μmol), and 90°C (0.92 μmol), while pre-incubation for 1 h at 100°C resulted in the complete loss of activity (Figure 4). Fsa16295Glu was active across the entire tested pH range of 5.5–11.0 and optimally active between 6.5 and 10.0 (Supplementary Figure S5). Using a Lineweaver–Burk plot (*R*^2^ = 0.9907) and assuming 100% enzyme activity with Fsa16295Glu at a concentration of 141 μg/mL, V_max_, K_M_, and *k*_cat_/K_M_ were calculated as 133 μM/min, 0.0520 μM, and 1.82 × 10^7^ s^−1^ M^−1^_,_ respectively, using CM-curdlan (Supplementary Figure S6). V_max_, K_M_, and *k*_cat_/K_M_ were calculated as 625 μM/min, 15.7 μM, and 2.83 × 10^5^ s^−1^ M^−1^_,_ respectively, using oat β-glucan (Supplementary Figure S7). These K_M_ and *k*_cat_/K_M_ values reflect greater specificity and catalytic efficiency, respectively, with β-1,3-glycosidic linkages in CM-curdlan over the alternating β-1,3/1,4-glycosidic linkages in oat β-glucan.

### 3.3 Phylogenetic analysis of Fsa16295Glu within family GH50

**Figure 5:**
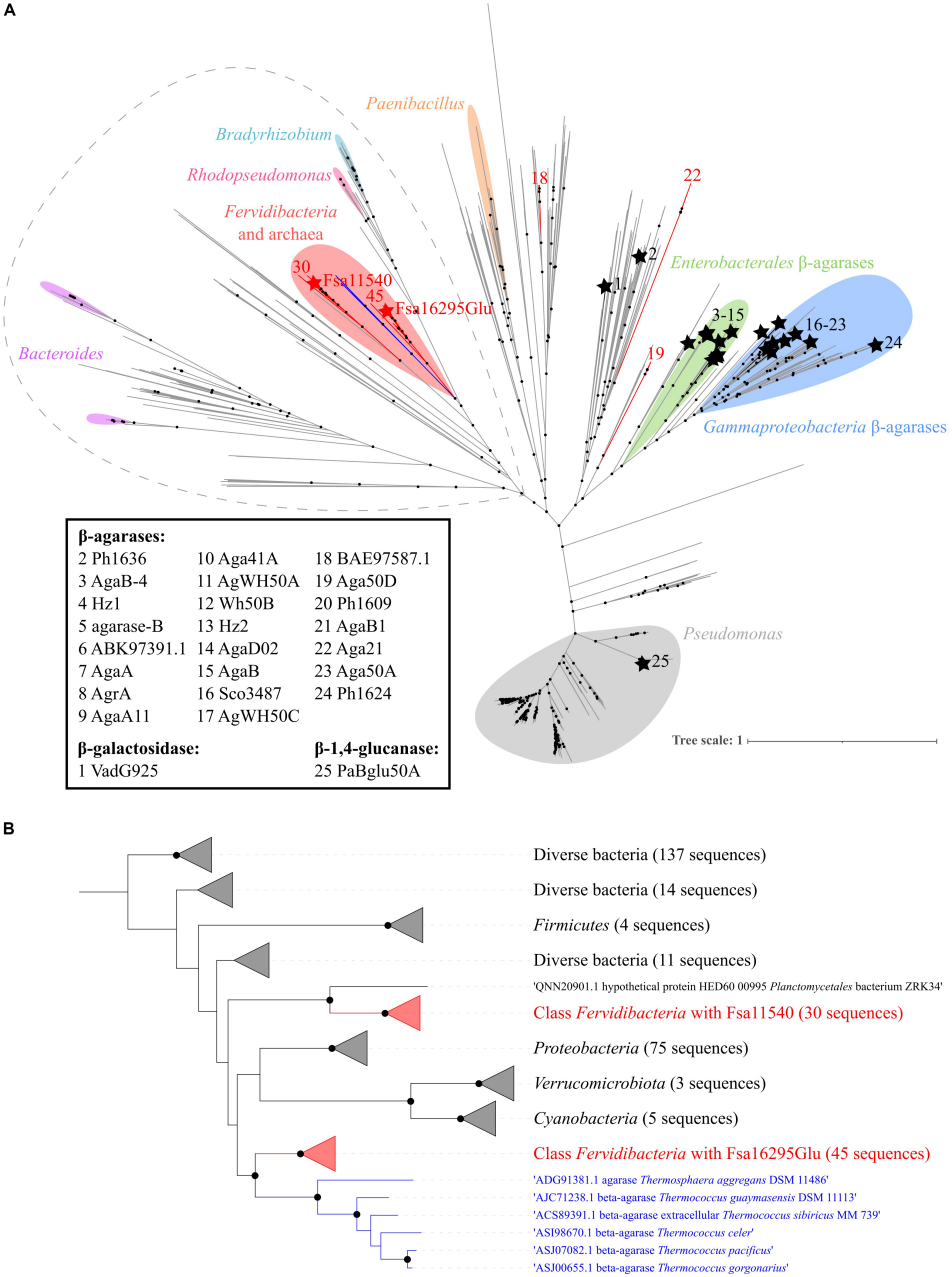
Phylogenetic analysis of GH50. **(A)** An approximate maximum likelihood analysis of GH50 using FastTree and SH-aLRT branch support. Sequences from *Fervidibacteria* are denoted by red branches. Fsa16295Glu and Fsa11540 are indicated by red stars, while other characterized GH50s are indicated by black branches, black stars, and an identifying number that corresponds to their protein names or GenBank accession numbers if no name was listed. Clusters from the same genera are marked with colored highlights, and characterized GH50s are clustered by their lowest common taxonomic rank. The sequences included in panel B are circled by a dashed gray line. Well-supported branches (SH-aLRT ≥0.9) are indicated by a black dot. The scale bar shows the number of amino acid changes per site. **(B)** A maximum likelihood analysis of the Fsa16295Glu lineage and its closest relatives (circled in dashed grey outline) using SH-aLRT and UFBoot branch support. Sequences are clustered into five *Fervidibacteria* clades (red; the number of sequences is numbered at the tips), the archaeal GH50s (blue), and related GH50s by taxonomic rank. Well-supported branches (SH-aLRT ≥0.9 and UFBoot ≥95%) are indicated by a black dot. The scale bar shows the number of amino acid changes per site.

Based on approximate maximum likelihood phylogenetic analyses of Fsa16295Glu, other GH50 enzymes from class *Fervidibacteria* gathered from related metagenome-assembled genomes (Nou et al., 2024), and all members of family GH50 within the CAZy database, Fsa16295Glu and homologs from other *Fervidibacteria* had a unique phylogenetic placement compared to other characterized GH50s (Figure 5A). *Fervidibacteria* GH50s were placed into five monophyletic clades, containing 18, 19, 22, 30, and 45 sequences, with Fsa16295Glu belonging to the largest clade. The 25 previously characterized GH50s included in this analysis belonged to *Pseudomonadota* (22 from *Agarivorans gilvus* WH0801, *Agarivorans* sp. AG17, *Agarivorans* sp. HZ105, *Agarivorans* sp. JA-1, *Agarivorans* sp. JAMB-A11, *Agarivorans* sp. QM38, *Alteromonas* sp. E-1, *Paraglaciecola hydrolytica* S66, *Pseudoalteromonas* sp. NJ21, *P. aeruginosa*, *Saccharophagus degradans* 2–40, *Thalassotalea agarivorans* BCRC 17492, *Vibrio* sp. CN41, *Vibrio* sp. PO-303); *Verrucomicrobiota* (one from *V. vadensis* ATCC BAA-548); *Bacillota* (one from *Paenibacillus agarexedens* BCRC 16000); and *Actinomycetota* (one from *Streptomyces coelicolor* A3(2)). Most *Pseudomonadota* sequences clustered into two groups of enzymes according to their lowest common taxonomic rank: β-agarases from *Enterobacterales* (genera *Agarivorans* and *Vibrio*) and β-agarases from a broader group of *Gammaproteobacteria* (including the order *Enterobacterales*_A and the *Pseudomonadales* genera *Alteromonas*, *Paraglaciecola*, *Pseudoalteromonas*, *Pseudomonas*, *Saccharophagus*, and *Thalassotalea*.) Consistent with their distinct activities from the other characterized enzymes, VadG925 (homolog 1 in Figure 5A and Supplementary Table S1) and PaBglu50A (homolog 25 in Figure 5A and Supplementary Table S1) were distant from the well-characterized GH50 groups. The maximum likelihood analysis showed that the *Fervidibacteria* GH50 group that Fsa16295Glu belongs to was most closely related to six archaeal GH50s from two unrelated archaea, *Thermosphaera aggregans* in the *Thermoproteota,* and several species of *Thermococcus* in the Methanobacteriota_B (Figure 5B) (Taxonomic names are listed as in GTDB.) Along with sequences from *Fervidibacteria*, these archaeal enzymes were the only GH50s in this analysis from thermophiles. The monophyly of *Fervidibacteria* homologs in this lineage suggests vertical evolution of the enzymes related to Fsa16295Glu in the class *Fervidibacteria*, with horizontal transfers to different thermophilic archaea, either independently, or horizontal transfer to *T. aggregans*, and then from *T. aggregans* to *Thermococcus.* The second *“F. sacchari”* GH50 enzyme, Fsa11540, belonged to a second major *Fervidibacteria* clade, implying that the two paralogous GH50s of *“F. sacchari”* represent distinct enzymes with potentially different activities or regulation. Organisms encoding GH50s that were related to Fsa16295Glu and Fsa11540 included members of the *Pseudomonadota* (75)*, Cyanobacteriota* (5)*, Bacillota* (4), and *Verrucomicrobiota* (3).

## 4 Discussion

Prior to this study, characterized GH50s have been almost exclusively classified as β-agarases, with the notable exceptions being a β-galactosidase and a β-1,3-glucanase (Temuujin et al., 2012a; Yi et al., 2018). Our study, however, reveals a GH50 β-1,3-endoglucanase that is phylogenetically distant from the previously characterized β-1,3-glucanase, PaBglu50A. Although Fsa16295Glu could degrade agarose, suggesting some β-agarase activity, its hydrolysis of CM-curdlan and oat β-glucan released considerably more reducing sugars. Thus, Fsa16295Glu is the first known GH50 to degrade substrates with both galactose units, found in agarose, and glucose units, found in CM-curdlan, oat β-glucan, and laminarin. Fsa16295Glu furthermore deviates from other GH50s through its high temperature optimum of 80°C, a marked increase over the second-highest temperature optimum in GH50: 65°C of AgrA from *Agarivorans* sp. AG17 (Nikapitiya et al., 2010). Overall, Fsa16295Glu is a functionally distinct GH50 and the first known hyperthermophilic enzyme within the family GH50.

Compared with other characterized GH50s, Fsa16295Glu has a relatively high *k*_cat_/K_M_ of 1.82 × 10^7^ s^−1^ M^−1^. GH50 β-agarase *k*_cat_/K_M_ values range from 8.1 × 10^2^ to 5.7 × 10^7^ s^−1^ M^−1^, with Fsa16295Glu falling just under that of Sco3487 from *Streptomyces coelicolor* A3(2), which was described as having a high catalytic efficiency (5.7 × 10^7^ s^−1^ M^−1^) (Fu et al., 2008; Temuujin et al., 2012b; Chen et al., 2018). The lower K_M_ of Fsa16295Glu toward CM-curdlan (0.0520 μM) compared with oat β-glucan (15.7 μM) indicates preferential hydrolysis of β-1,3-glucans like CM-curdlan. The inability of Fsa16295Glu to degrade CMC suggests it cannot cleave β-1,4-glycosidic linkages connecting glucose, which is supported by the partial (65.6%) hydrolysis of oat β-glucan, indicating hydrolysis of β-1,3-, but not β-1,4-glycosidic linkages. Furthermore, the near-complete (79.2%) hydrolysis of CM-curdlan further supports the catalysis of β-1,3-glycosidic linkages. That CM-curdlan is not more thoroughly degraded despite the ubiquity of β-1,3-glycosidic linkages may be caused by the presence of carboxymethyl groups on every third glucose, potentially limiting access by Fsa16295Glu to one or both sides of the carboxymethylated residue. Thus, possible degradation products include glucose and CM-glucose, with some Glc (β1,3)CM-Glc or CM-Glc (β1,3)Glc. Side chain inhibition is supported by the comparatively poor (35.0%) hydrolysis of laminarin and the inability of Fsa16295Glu to hydrolyze yeast β-glucan. Laminarin from stramenopiles, such as that used in this study, contains a β-1,6-linked glucose side chain on every third residue (Chen et al., 2021), which may further limit access by Fsa16295Glu. Additionally, the inability of Fsa16295Glu to degrade yeast β-glucan suggests that side chain inhibition may be exacerbated by chain length, as yeast β-glucan frequently contains side chains with more than one glucose (Liu et al., 2021). Although degradation of β-1,4-glucan was not observed, activity on agarose suggests some cleavage of β-1,4-glycosidic linkages connecting galactose residues. Due to the microbial origin of some β-1,3-glucans (Mudgil, 2017; Paudel et al., 2021), we speculate that bacteria found *in situ* are the natural source of β-1,3-glucan. Various bacteria produce curdlan, including *Agrobacterium*, *Rhizobium*, and *Cellulomonas* (Gao et al., 2020), but none are known to be thermophilic, obscuring the possible source of curdlan, if it exists, in the geothermal spring environment of *“F. sacchari”* and other *Fervidibacteria*. One possible source of β-1,3-glucan is the photosynthetic mats found at the fringes of the Great Boiling Spring (GBS) that were originally used to enrich *“F. sacchari”* (Nou et al., 2024). Glucose is found in more than 90% of polysaccharides released by *Cyanobacteriota*, which are prominent constituents of photosynthetic mats, and is often the most abundant monosaccharide (De Philippis and Vincenzini, 1998; George et al., 2023). However, photosynthetic mats at GBS are located ~1–2 meters away from higher temperature sediments hosting abundant populations of *“F. sacchari,”* and the polysaccharide composition of these mats has not been explored. Further study is needed to explore the specific β-1,3-glucans depolymerized by Fsa16295Glu and their sources in geothermal springs.

Fsa16295Glu has a unique phylogenetic position relative to other characterized GH50s. Aside from other GH50s from *Fervidibacteria*, Fsa16295Glu is most closely related to a subset of six GH50s encoded by several *Thermococcus* species and *T. aggregans*. Given the presence of homologs of Fsa16295Glu in most available *Fervidibacteria* genomes and the distant relationship between *Fervidibacteria*, *Thermococcus*, and *Thermosphaera*, we infer that this enzyme passed through horizontal gene transfer from *Fervidibacteria* to these archaea, which are common co-inhabitants in geothermal environments. The lower activity of Fsa16295Glu on agarose coincides with substrate availability, as all agarolytic GH50s are from marine environments, where agar is a major constituent of red algae (Yun et al., 2021). The presence of weak agarase activity in Fsa16295Glu suggests that β-agarase and β-1,3-glucanase activities may have been ancestral in GH50, but adaptation to polysaccharides in different environments was selected for divergent activities. Further characterization of diverse GH50s and ancestral character state reconstructions could offer insight into the validity of this hypothesis.

The assignment of GHs into families based on amino acid sequences is somewhat limited by the breadth of known GH families and the limited knowledge of their activities. However, new GH families are often discovered, as recently as the discovery of GH174 by Liu et al. (2023). Because of this, the functional and phylogenetic novelty of Fsa16295Glu prompted us to consider whether Fsa16295Glu should be assigned to a new GH family. However, the presence of two conserved GH-A glutamic acid residues in Fsa16295Glu and the relatively high amino acid identity to canonical GH50s (29.91–33.26%) suggests that Fsa16295Glu correctly falls within GH50 but should be distinguished from other characterized GH50s. Viborg et al. (2019) previously proposed a roadmap for identifying GH subfamilies based on combinations of phylogenetic coherence and functional similarity. Because of their close phylogenetic relationship and shared agarolysis, we propose GH50s from the clade containing characterized *Enterobacterales* and *Gammaproteobacteria* β-agarases be designated as subfamily GH50_1. Similarly, we propose the β-1,3-glucanase PaBglu50A from the *Pseudomonas* clade be designated as the first characterized member of GH50_2. Finally, because of its β-1,3-glucanase activity, 80°C optimum, and distant relationship to other characterized GH50s, we propose Fsa16295Glu be designated as the first characterized representative of GH50_3.

## Data availability statement

The datasets presented in this study can be found in online repositories. The names of the repository/repositories and accession number(s) can be found in the article/Supplementary material.

## Author contributions

JC: Conceptualization, Data curation, Formal analysis, Funding acquisition, Investigation, Methodology, Supervision, Visualization, Writing – original draft, Writing – review & editing. NT: Writing – review & editing, Data curation, Formal analysis, Funding acquisition, Investigation, Methodology. AC: Writing – review & editing, Data curation, Formal analysis, Funding acquisition, Investigation, Methodology. MP: Conceptualization, Data curation, Formal analysis, Investigation, Methodology, Supervision, Visualization, Writing – review & editing. SB: Writing – review & editing, Data curation, Formal analysis, Investigation, Methodology. NN: Conceptualization, Data curation, Funding acquisition, Writing – review & editing. RM: Writing – review & editing, Conceptualization, Data curation, Methodology. MH-S: Writing – review & editing, Data curation, Project administration. IB: Conceptualization, Data curation, Methodology, Project administration, Supervision, Writing – review & editing. J-FC: Writing – review & editing, Conceptualization, Data curation, Methodology, Project administration, Supervision. MH: Conceptualization, Methodology, Resources, Writing – review & editing. PB: Methodology, Writing – review & editing. NS: Writing – review & editing, Funding acquisition, Methodology. KV: Funding acquisition, Methodology, Writing – review & editing. BH: Supervision, Writing – original draft, Writing – review & editing, Conceptualization, Funding acquisition, Project administration, Resources.
