# Supplementary material for: Biochemical characterization of Fsa16295Glu from “Fervidibacter sacchari,” the first hyperthermophilic GH50 with β-1,3-endoglucanase activity and founding member of the subfamily GH50_3
Source: Front Microbiol. 2024 Apr 25;15:1355444. doi: 10.3389/fmicb.2024.1355444 (PMC11079163; doi:10.3389/fmicb.2024.1355444)
Supplement: Supplementary file 1 [file Data_Sheet_1.pdf]

```
Fsa16295Glu/1-454    1 M------------------------------------------------    1
Fsa11540/1-1154      1 MR-KL--------------------------------------------    4
Aga50D/1-793         1 MG-----------------------------------------------    2
AgaA/1-995           1 MKIKFLSAAIAASLALPLSAATLVTSFEEADYSSSENNAEFLEVSGDAT   49
AgrA/1-995           1 MKIKFLSAAIAASLALPLSAATLVTSFEEADYSSSENNTEFLEVSGDAT   49
PaBglu50A/1-749      1 MI-----------------------------------------------    2
VadG925/1-1425       1 MS-KLLHLYV-----------ILGTIF----------------------   15

Fsa16295Glu/1-454    2 --------------------------------SW---------------    3
Fsa11540/1-1154      5 --------------------------------AWVVGAI----------   11
Aga50D/1-793         3 -------AIGGLVKINISFIPLFVI-----SASIFIGAC----------   29
AgaA/1-995          50 SEVSTEQATDGNQSIKASFDAAFKPMVVWNWASWNWGAEDVMSVDVVNP   98
AgrA/1-995          50 SEVSTEQATDGNQSIKASFDAAFKPMVVWNWGSWNWGAEDVMSVDVVNP   98
PaBglu50A/1-749      3 ------------------------------RSRWHLPLLL---------   12
VadG925/1-1425      16 -------------SLEAAPIPANPP-----VTAW---------------   31

Fsa16295Glu/1-454      -------------------------------------------------
Fsa11540/1-1154     12 ----AAFAL----------WL-----------ASANAHQPFRFS-----   30
Aga50D/1-793        30 -----------------------NSSKLESGVDSNNISPVMLF------   49
AgaA/1-995          99 NDTDVTFAIKLIDSDILPDWVDESQTSLDYFTVSANTTQTFSFNLNGGN  147
AgrA/1-995          99 NDTDVTFAIKLIDSDILPDWVDESQTSLDYFTVSANTTQTFSFNLNGGN  147
PaBglu50A/1-749     13 --GLLAVATPLAASDI----------------------QQVLF------   31
VadG925/1-1425         -------------------------------------------------

Fsa16295Glu/1-454      -------------------------------------------------
Fsa11540/1-1154     31 ------DDFSK------------------------------YPAGSIGE   43
Aga50D/1-793        50 -------DFENDQV-----------PSNIHFLNAR--------------   66
AgaA/1-995         148 EFQTHGENFSKDKVIGVQFMLSENDPQVLYFDNIMVDGETVTPPPSDGA  196
AgrA/1-995         148 EFQTHGENFSKDKVIGVQFMLSENDPQVLYFDNIMVDGETVTPPPSDGA  196
PaBglu50A/1-749     32 -------NFVK--------------PMAVVGITLE---DADLP------   50
VadG925/1-1425      32 -------------------------PQLLRQGKLQ--------------   41

Fsa16295Glu/1-454      -------------------------------------------------
Fsa11540/1-1154     44 PNWEVTDI--------------------GFEIRNGTMHAEVVAGRGYAI   72
Aga50D/1-793        67 -----------ASIETYTGING--EPSKGLKL---AMQSKQHSYTGLAI   99
AgaA/1-995         197 VNTQTAPVATLAQIEDFETIPDYLRPDGGVNV---STTTEIVTKGAAAM  242
AgrA/1-995         197 VNTQTAPVATLAQIEDFETIPDYLRPDGGVNV---STTTEIVTKGAAAM  242
PaBglu50A/1-749     51 -----------------------------------SATAEATPEGDILR   64
VadG925/1-1425      42 -----------------------------------GSPESSFACDGEML   55

Fsa16295Glu/1-454      -------------------------------------------------
Fsa11540/1-1154     73 LKVAPMGRSVTVEATLTVHRAISTGWKIAGVG----IFVD-----ERNY  112
Aga50D/1-793       100 V--------------------PEQPWDWSEFT-SASLYFDIVSVGDHST  127
AgaA/1-995         243 AAEFTAGWNGLV---------FAGTWNWAELGEHTAVAVDVSNNSDSNI  282
AgrA/1-995         243 AAEFTAGWNGLV---------FAGTWNWAELGEHTAVAVDVSNTSDSNI  282
PaBglu50A/1-749     65 RVTFSPAQRPTLRMSP-----ALGRWDWSAAD-YVSLRIQNAMSWDMTL  107
VadG925/1-1425      56 VFSVPAGRGGSM---------TFPVTEPSAGESFTRLELELENRGDTGT   95

Fsa16295Glu/1-454      -------------------------------------------------
Fsa11540/1-1154    113 WHIALVESPDNQGK----RHFAELHQMLDGVWLSDVQDPTRLTTEEETG  157
Aga50D/1-793       128 QFYLDVTD--QNGAV-FTRSIDIPVGKMQSYYAKLSGHDLEVPDSGDVN  173
AgaA/1-995         283 WLYSRIEDVNSQGET-ATRGVLVKAGESKTIYTSLNDNPSLLTQDERVS  330
AgrA/1-995         283 WLYSRIEDVNSQGET-ATRGVLVKAGESKTIYTSLNDNPSLLTQDERVS  330
PaBglu50A/1-749    108 EVAIE----GEQGAPGLQASIELPAGPPQTLLVPLRAVSPE--------  144
VadG925/1-1425      96 GFTIRLRS--ADGRA-VTVHCRIEAGQCKQAVAPMPRDRKTLETR----  137

Fsa16295Glu/1-454    4 -----------TR------------------------------------    5
Fsa11540/1-1154    158 GFDWEYGRPYRLR---------------------IALSKERV------I  179
Aga50D/1-793       174 DLNLASG----LRSNPPTWTSDDRQFVWMWGV--KNLDLSGIAKISL--  214
AgaA/1-995         331 ----ALG----LRDIPADPMSAQN----GWGD-FVALDKSQITAIRYFI  366
AgrA/1-995         331 ----ALG----LRDIPADPMSAQN----GWGD-FVALDKSQITAIRYFI  366
PaBglu50A/1-749    145 ----ALG----MRAGPPMPQMVEGQRVLLAPRVEGSLDRARVGALSL--  183
VadG925/1-1425     138 ---YAAQ----LRDFPKMAGLPGG----LFEN-WESVDAGDLEAVTL--  172
```

```
Fsa16295Glu/1-454    6 -------------------------------------REFVKLVGFATT  17
Fsa11540/1-1154    180 GEVFDAD-------GKLRYRCVRRLDKRAVTFG----RPILTCGGFVAT 217
Aga50D/1-793       215 -SVQSAMHDKTVIIDNIRIQPNPPQDENFLV-------------GLVDE 249
AgaA/1-995         367 GELASGETSQTLVFDNMRVIKDLNHESAY--------------AEMTDA 401
AgrA/1-995         367 GELASGETSQTLVFDNMRVIKDLNHESAY--------------AEMTDA 401
PaBglu50A/1-749    184 -SLRSPQAPQSILLGRFGIRTGRAVERSILT-------------GLIDR 218
VadG925/1-1425     173 -EFPEAAAPLTLRIGQVRF--SHPAAPSLYAAAPERF------FPFIDR 212

Fsa16295Glu/1-454   18 FAG------------------------ASCRSEGQEEAKFCRYGGWLAM  42
Fsa11540/1-1154    218 FDD-------------------VKAEVNEIV--SEPKRERKTYPPFVSR 245
Aga50D/1-793       250 FGQNAKVDYKGKIHSLEELHAARDVELAELD--GKPMPSRSKFGGWLAG 296
AgaA/1-995         402 MGQNNLVTYAGKVASKEELAKLSDPEMAALG----ELTNRNMYGGNPDS 446
AgrA/1-995         402 MGQNNLVTYAGKVASKEELAKLSDPEMAVLG----ELTNRNMYGGNPDS 446
PaBglu50A/1-749    219 YGQYSRADWPEKIRSDEQLRSAYAAEAAQLRDWERQTPARDRFGGLLGG 267
VadG925/1-1425     213 YGQYIHEGWPGKVKSDQELKLAWEAEKNDLAA-HRRPASFSKFGGWREG 260

Fsa16295Glu/1-454   43 KR-------KATGFFRTEQVNGVWWFVDPDGHLFISKGVNHVSFGGDYC  84
Fsa11540/1-1154    246 PSPHAPRPVKGTGFFRTQQINGIWWLIDPNGHPTLSIGTDHVNYFVHWC 294
Aga50D/1-793       297 PK------LKATGYFRTEKINGKWMLVDPEGYPYFATGLDIIRLSNSST 339
AgaA/1-995         447 SPATDCVLATPASFNACKDADGNWQLVDPAGNAFFSTGVDNIRLQDTYT 495
AgrA/1-995         447 SPTTDCVLATPASFNACKDADGNWQLVDPAGNAFFSTGVDNIRLQDTYT 495
PaBglu50A/1-749    268 PV------FEASGFFRTEKRGGRWWLVTPEGHPFWSLGVNAVTADGSRT 310
VadG925/1-1425     261 PR------FKATGSFYPKKYDGKWYLADPEGYLFWSNGINCVRAETYGT 303

Fsa16295Glu/1-454      -------------------------------------------------
Fsa11540/1-1154        -------------------------------------------------
Aga50D/1-793       340 MTGYDYDQATVAQRSADDVTPEDSKGLMAVSEKSFATRHLASPTRAAMF 388
AgaA/1-995         496 MTGVSSDAES------------------------------ESALRQSMF 514
AgrA/1-995         496 MTGVSSDAES------------------------------ESALRQSMF 514
PaBglu50A/1-749    311 YV----------------------------------------EGREPMF 319
VadG925/1-1425     304 LV----------------------------------------EGRERFF 312

Fsa16295Glu/1-454   85 ---------------------------------PALGYSPYNRNVQAKY 100
Fsa11540/1-1154    295 ---------------------------------EKLGYAPYHENVKRKY 310
Aga50D/1-793       389 NWLPDYDHPLANHYNYRRSAHSGPLK-------RGEAYSFYSANLERKY 430
AgaA/1-995         515 TEIPS--DYVNENYG---PVHSGPVS-------QGQAVSFYANNLITRH 551
AgrA/1-995         515 TEIPS--DYVNENYG---PVHSGPVS-------QGQAVSFYANNLITRH 551
PaBglu50A/1-749    320 AELPAEGEPLAAFFGEG-DDRRGVAAQAGRRFGHGRWFDFLGANRQRIA 367
VadG925/1-1425     313 AGLPEAGTPCSAFFHNT-ERYGG----------KHLAYNFAAANLLRKY 350

Fsa16295Glu/1-454  101 GSE------EKWAE----ATAKRLREWGFNTIGAWS-SRSLFKL----M 134
Fsa11540/1-1154    311 GSE------EAWAK----EAVKRLLSWNFNVLGANNSVKARYQG----L 345
Aga50D/1-793       431 GETYPGSYLDKWRE----VTVDRMLNWGFTSLGNWT-DPAYYDN--NRI 472
AgaA/1-995         552 ASE------DVWRD----ITVKRMKDWGFNTLGNWT-DPALYAN--GDV 587
AgrA/1-995         552 ASE------DVWRD----ITVKRMKDWGFNTLGNWT-DPALYAN--GDV 587
PaBglu50A/1-749    368 PQASADQLAGEWRR----RTLERLSAWGFNSLGNWS-DPALAAQ--ARM 409
VadG925/1-1425     351 GPD--------WRDLHLGLTGKRLASWGFNTVGNWS-D---YAGKIADF 387

Fsa16295Glu/1-454  135 PYTVILNMGA--------RVGADW----LKGSFPDVFSPKFRQVLDEIA 171
Fsa11540/1-1154    346 AHTEFLAFGS---DF---ASTADIVPKVHWTGFPDVFDPRFERFCDLRA 388
Aga50D/1-793       473 PFFANGWVIG---DFKTVSSGAD-----FWGAMPDVFDPEFKVRAMETA 513
AgaA/1-995         588 PYVANGWSTSGADRLPVKQIGSG-----YWGPLPDPWDANFATNAATMA 631
AgrA/1-995         588 PYVANGWSTSGADRLPVKQIGSG-----YWGPLPDPWDANFATNAATMA 631
PaBglu50A/1-749    410 PYSLPLSIAG---DYATVSSGFD-----WWGAMPDPFDPRFAMAAERVI 450
VadG925/1-1425     388 PYVLTLGHACR-------SSG---------GWRDDPFDPAHRENIEQAL 420

Fsa16295Glu/1-454  172 AR-ECAPRKNDPLLVGYFTDNELRW-GPDWRSP-----RHLLDDYLLLL 213
Fsa11540/1-1154    389 KR-RCAPNKDDPWLLGYFLDNELEWWGKSGR-------PWGMAEEAWKK 429
Aga50D/1-793       514 RV-VSEEIKNSPWCVGVFIDNEKSF-GRPDSDKA----QYGIPIHTLGR 556
AgaA/1-995         632 AEIKAQVEGNEEYLVGIFVDNEMSW-GNVTDVEGSRYAQTLAVFNTDGT 679
AgrA/1-995         632 AEIKAQVEGNEEYLVGIFVDNEMSW-GNVTDVEGSRYAQTLAVFNTDGT 679
PaBglu50A/1-749    451 AI-AARDHRDDPWLLGYYADNELAWAGRDGSAQA----RYGLAFGALTL 494
VadG925/1-1425     421 QSGRYDAAIRDPYCIGFFSGNELPW-SDPVTYAG-----------NLLR 457
```

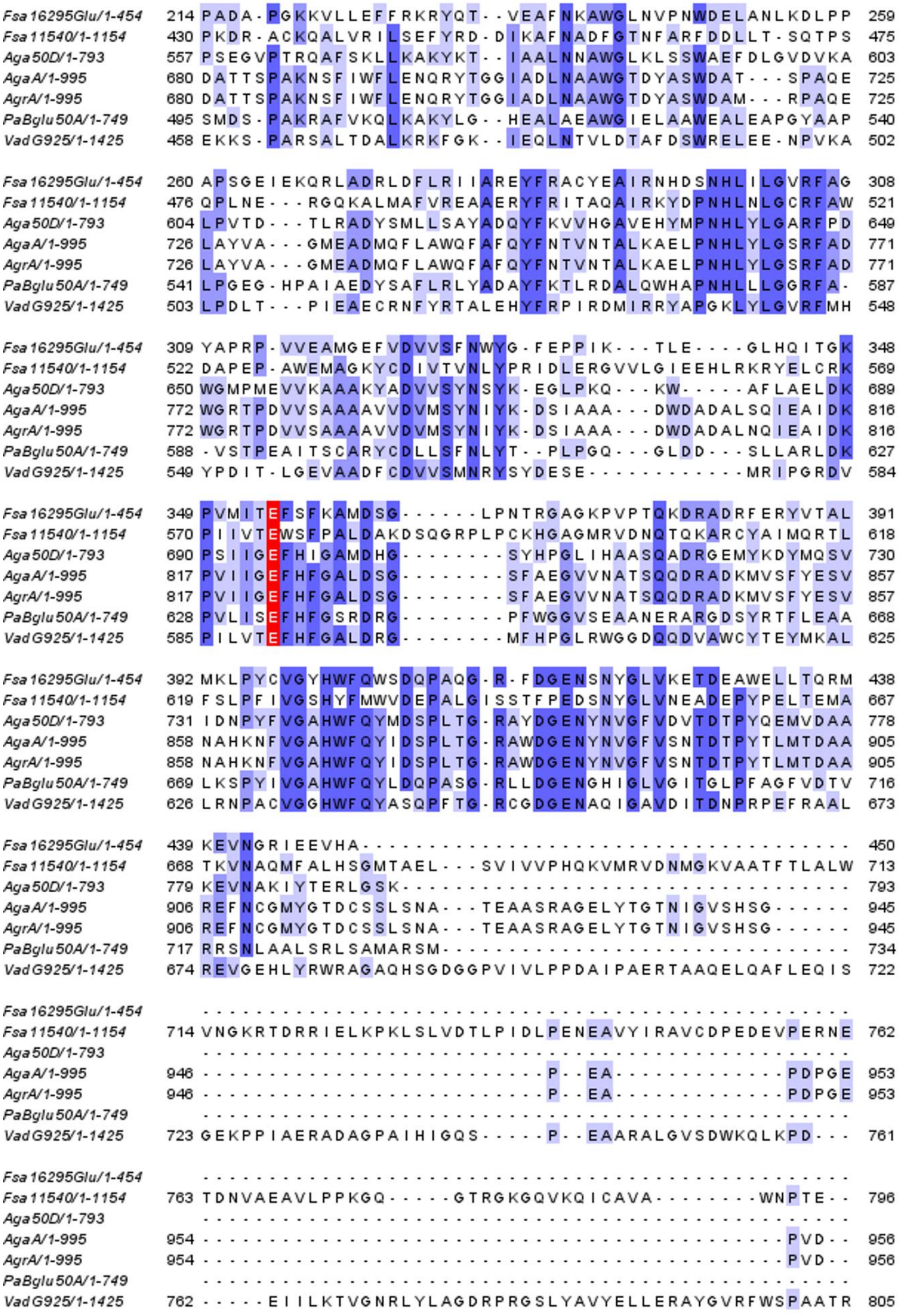
Fsa16295Glu/1-454 214 PADA-PGKKVLLEFFRKRYQT--VEAFNKAWGLNVPNWDELANLKDLPP 259
Fsa11540/1-1154 430 PKDR-ACKQALVRILSEFYRD-DIKAFNADFGTNFARFDDLLT-SQTPS 475
Aga50D/1-793 557 PSEGVPTRQAFSKLLKAKYKT--IAALNNAWGLKLSSWAEFDLGVDVKA 603
AgaA/1-995 680 DATTSPAKNSFIWFLENQRYTGGIADLNAAWGTDYASWDAT---SPAQE 725
AgrA/1-995 680 DATTSPAKNSFIWFLENQRYTGGIADLNAAWGTDYASWDAM---RPAQE 725
PaBglu50A/1-749 495 SMDS-PAKRAFVKQLKAKYLG--HEALAEAWGIELAAWEALEAPGYAAP 540
VadG925/1-1425 458 EKKS-PARSALTDALKRKFGK--IEQLNTVLDTAFDSWRELEE-NPVKA 502
Fsa16295Glu/1-454 260 APSGEIEKQRLADRLDFLRIIAREYFRACYEAIRNHDSNHLILGVRFAG 308
Fsa11540/1-1154 476 QPLNE---RGQKALMAFVREAAERYFRITAQAIRKYDPNHLNLGCRFAW 521
Aga50D/1-793 604 LPVTD---TLRADYSMLLSAYADQYFKVVHGAVEHYMPNHLYLGARFPD 649
AgaA/1-995 726 LAYVA---GMEADMQFLAWQFAFQYFNTVNTALKAELPNHLYLGSRFAD 771
AgrA/1-995 726 LAYVA---GMEADMQFLAWQFAFQYFNTVNTALKAELPNHLYLGSRFAD 771
PaBglu50A/1-749 541 LPGEG-HPAIAEDYSAFLRLYADAYFKTLRDALQWHAPNHLLLGGRFA- 587
VadG925/1-1425 503 LPDLT---PIEAECRNFYRTALEHYFRPIRDMIRRYAPGKLYLGVRFMH 548
Fsa16295Glu/1-454 309 YAPRP-VVEAMGEFVDVVSFNWYG-FEPPIK---TLE----GLHQITGK 348
Fsa11540/1-1154 522 DAPEP-AWEMAGKYCDIVTVNLYPRIDLERGVVLGIEEHLRKRYELCRK 569
Aga50D/1-793 650 WGMPMEVVKAAAKYADVVSYNSYK-EGLPKQ---KW-----AFLAELDK 689
AgaA/1-995 772 WGRTPDVVSAAAAVVDVMSYNIYK-DSIAAA---DWDADALSQIEAIDK 816
AgrA/1-995 772 WGRTPDVVSAAAAVVDVMSYNIYK-DSIAAA---DWDADALNQIEAIDK 816
PaBglu50A/1-749 588 -VSTPEAITSCARYCDLLSFNLYT--PLPGQ---GLDD---SLLARLDK 627
VadG925/1-1425 549 YPDIT-LGEVAADFCDVVSMNRYSYDESE------------MRIPGRDV 584
Fsa16295Glu/1-454 349 PVMITEFSFKAMDSG------LPNTRGAGKPVPTQKDRADRFERYVTAL 391
Fsa11540/1-1154 570 PIIVTEWSFPALDAKDSQGRPLPCKHGAGMRVDNQTQKARCYAIMQRTL 618
Aga50D/1-793 690 PSIIGEFHIGAMDHG--------SYHPGLIHAASQADRGEMYKDYMQSV 730
AgaA/1-995 817 PVIIGEFHFGALDSG--------SFAEGVVNATSQQDRADKMVSFYESV 857
AgrA/1-995 817 PVIIGEFHFGALDSG--------SFAEGVVNATSQQDRADKMVSFYESV 857
PaBglu50A/1-749 628 PVLISEFHFGSRDRG--------PFWGGVSEAANERARGDSYRTFLEAA 668
VadG925/1-1425 585 PILVTEFHFGALDRG--------MFHPGLRWGGDQQDVAWCYTEYMKAL 625
Fsa16295Glu/1-454 392 MKLPYCVGYHWFQWSDQPAQG-R-FDGENSNYGLVKETDEAWELLTQRM 438
Fsa11540/1-1154 619 FSLPFIVGSHYFMWVDEPALGISSTFPEDSNYGLVNEADEPYPELTEMA 667
Aga50D/1-793 731 IDNPYFVGAHWFQYMDSPLTG-RAYDGENYNVGFVDVTDTPYQEMVDAA 778
AgaA/1-995 858 NAHKNFVGAHWFQYIDSPLTG-RAWDGENYNVGFVSNTDTPYTLMTDAA 905
AgrA/1-995 858 NAHKNFVGAHWFQYIDSPLTG-RAWDGENYNVGFVSNTDTPYTLMTDAA 905
PaBglu50A/1-749 669 LKSPYIVGAHWFQYLDQPASG-RLLDGENGHIGLVGITGLPFAGFVDTV 716
VadG925/1-1425 626 LRNPACVGGHWFQYASQPFTG-RCGDGENAQIGAVDITDNPRPEFRAAL 673
Fsa16295Glu/1-454 439 KEVNGRIEEVHA------------------------------------- 450
Fsa11540/1-1154 668 TKVNAQMFALHSGMTAEL---SVIVVPHQKVMRVDNMGKVAATFTLALW 713
Aga50D/1-793 779 KEVNAKIYTERLGSK---------------------------------- 793
AgaA/1-995 906 REFNCGMYGTDCSSLSNA---TEAASRAGELYTGTNIGVSHSG------ 945
AgrA/1-995 906 REFNCGMYGTDCSSLSNA---TEAASRAGELYTGTNIGVSHSG------ 945
PaBglu50A/1-749 717 RRSNLAALSRLSAMARSM------------------------------- 734
VadG925/1-1425 674 REVGEHLYRWRAGAQHSGDGGPVIVLPPDAIPAERTAAQELQAFLEQIS 722
Fsa16295Glu/1-454 ---
Fsa11540/1-1154 714 VNGKRTDRRIELKPKLSLVDTLPIDLPENEAVYIRAVCDPEDEVPERNE 762
Aga50D/1-793 ---
AgaA/1-995 946 --------------------------P--EA-------------PDPGE 953
AgrA/1-995 946 --------------------------P--EA-------------PDPGE 953
PaBglu50A/1-749 ---
VadG925/1-1425 723 GEKPPIAERADAGPAIHIGQS-----P--EAARALGVSDWKQLKPD--- 761
Fsa16295Glu/1-454 ---
Fsa11540/1-1154 763 TDNVAEAVLPPKGQ-----GTRGKGQVKQICAVA--------WNPTE-- 796
Aga50D/1-793 ---
AgaA/1-995 954 --PVD-- 956
AgrA/1-995 954 --PVD-- 956
PaBglu50A/1-749 ---
VadG925/1-1425 762 -----EIILKTVGNRLYLAGDRPRGSLYAVYELLERAYGVRFWSPAATR 805

```
Fsa16295Glu/1-454        -------------------------------------------------
Fsa11540/1-1154    797   ---QTLRNVPVSVP--LPPAL-SSAEDIFVSD-------AKGNLLPSQV   832
Aga50D/1-793             -------------------------------------------------
AgaA/1-995         957   ---------PPIDPP-TPPT-----------------------------   966
AgrA/1-995         957   ---------PPIDPP-TPPT-----------------------------   966
PaBglu50A/1-749    735   ---------PAVEP--LPPRE----------------------------   744
VadG925/1-1425     806   VPRASLQQLPRIDLRYAPPFEVRSVGSILTRNDFRYAVRLRHNGQSAFV   854

Fsa16295Glu/1-454        -------------------------------------------------
Fsa11540/1-1154    833   SDKLGSVTVLVKELKPYSAVTLWLSTTKGGRGSSRAEIPFAVHHAAKGE   881
Aga50D/1-793             -------------------------------------------------
AgaA/1-995         967   ----GGVT-----------------------------------------   970
AgrA/1-995         967   ----GGVT-----------------------------------------   970
PaBglu50A/1-749          -------------------------------------------------
VadG925/1-1425     855   PPEWGGLVTLLGNVHTFSE---YPDNALIPRDSGFREHP---EWFAERD   897

Fsa16295Glu/1-454        -------------------------------------------------
Fsa11540/1-1154    882   GYAIETPKLLLIKDEPDGDAFDRI--ILRDG--------GRGARDEEIE   920
Aga50D/1-793             -------------------------------------------------
AgaA/1-995               -------------------------------------------------
AgrA/1-995               -------------------------------------------------
PaBglu50A/1-749          -------------------------------------------------
VadG925/1-1425     898   GRRVPNGQLCLTNPELHRELVRRVRELLRAAPESRYISVSQNDNDDFCQ   946

Fsa16295Glu/1-454        -------------------------------------------------
Fsa11540/1-1154    921   LGSFTPLIWQVVAGQNLWVRPDRVERIEVVDVGPAILVVDIVFVKGRGT   969
Aga50D/1-793             -------------------------------------------------
AgaA/1-995               -------------------------------------------------
AgrA/1-995               -------------------------------------------------
PaBglu50A/1-749          -------------------------------------------------
VadG925/1-1425     947   CRSCAAFVEKHGNQSDLLLDTVNAVAAAVAEEFPATLVETLAYRYTRTP   995

Fsa16295Glu/1-454        -------------------------------------------------
Fsa11540/1-1154    970   GDEGRVITEV------GAGGKFEPLKAEPQPFRCAYRFTFFPDQPFFLS  1012
Aga50D/1-793             -------------------------------------------------
AgaA/1-995               -------------------------------------------------
AgrA/1-995               -------------------------------------------------
PaBglu50A/1-749          -------------------------------------------------
VadG925/1-1425     996   PATVKAAPNVLIRYCTFEADSFRPLTAK-------------QNRQFFRD  1031

Fsa16295Glu/1-454        -------------------------------------------------
Fsa11540/1-1154    1013  QCLWVENTGKQAWQWRGYYHYALSRIGGNSADDEVGGVNVPNYWLQFAS  1061
Aga50D/1-793             -------------------------------------------------
AgaA/1-995               -------------------------------------------------
AgrA/1-995               -------------------------------------------------
PaBglu50A/1-749          -------------------------------------------------
VadG925/1-1425     1032  LAAW-SRTAKQLMIWNYI-------------------ANFRKYYLPTPN  1060

Fsa16295Glu/1-454        -------------------------------------------------
Fsa11540/1-1154    1062  WR--DPKLRL--HYGVISLREDERLGFWFWKDEGGNQ------------  1094
Aga50D/1-793             -------------------------------------------------
AgaA/1-995         971   --------------------------------GGGGS------------   975
AgrA/1-995         971   --------------------------------GGGGS------------   975
PaBglu50A/1-749    745   --------------------------------DSAGS------------   749
VadG925/1-1425     1061  WRALGPDLRTFRSFGAISVYEQ---GAW----NGGGSVSDLPELRTWLT  1102

Fsa16295Glu/1-454        -------------------------------------------------
Fsa11540/1-1154    1095  -------------------------------------------HPDC--  1098
Aga50D/1-793             -------------------------------------------------
AgaA/1-995               -------------------------------------------------
AgrA/1-995               -------------------------------------------------
PaBglu50A/1-749          -------------------------------------------------
VadG925/1-1425     1103  ARLLWNPDLDTDTLIDEFLTGYYGPGADAVRTYMKLMNGAADRHPEVAG  1151
```

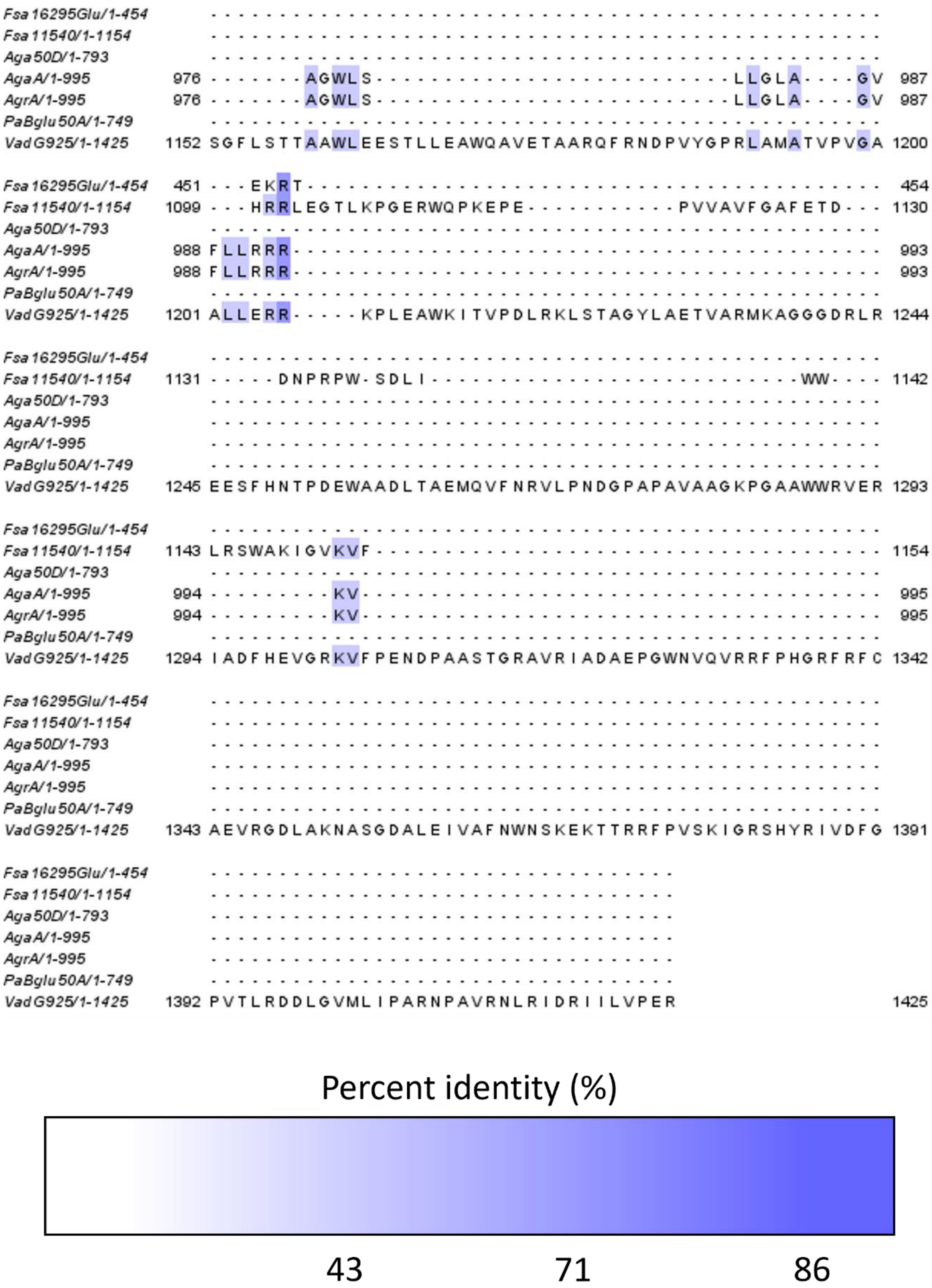
Fsa16295Glu/1-454
Fsa11540/1-1154
Aga50D/1-793
AgaA/1-995 976 --------AGWLS-------------------------LLGLA----GV 987
AgrA/1-995 976 --------AGWLS-------------------------LLGLA----GV 987
PaBglu50A/1-749
VadG925/1-1425 1152 SGFLSTTAAWLEESTLLEAWQAVETAARQFRNDPVYGPRLAMATVPVGA 1200
Fsa16295Glu/1-454 451 ---EKRT 454
Fsa11540/1-1154 1099 ---HRRLEGTLKPGERWQPKEPE-----------PVVAVFGAFETD--- 1130
Aga50D/1-793
AgaA/1-995 988 FLLRRR 993
AgrA/1-995 988 FLLRRR 993
PaBglu50A/1-749
VadG925/1-1425 1201 ALLERR----KPLEAWKITVPDLRKLSTAGYLAETVARMKAGGGDRLR 1244
Fsa16295Glu/1-454
Fsa11540/1-1154 1131 -----DNPRPW-SDLI-----WW---- 1142
Aga50D/1-793
AgaA/1-995
AgrA/1-995
PaBglu50A/1-749
VadG925/1-1425 1245 EESFHNTPDEWAADLTAEMQVFNRVLPNDGPAPAVAAGKPGAAWWRVER 1293
Fsa16295Glu/1-454
Fsa11540/1-1154 1143 LRSWAKIGVKVF 1154
Aga50D/1-793
AgaA/1-995 994 ---------KV 995
AgrA/1-995 994 ---------KV 995
PaBglu50A/1-749
VadG925/1-1425 1294 IADFHEVGRKVFPENDPAASTGRAVRIADAEPGWNVQVRRFPHGRFRFC 1342
Fsa16295Glu/1-454
Fsa11540/1-1154
Aga50D/1-793
AgaA/1-995
AgrA/1-995
PaBglu50A/1-749
VadG925/1-1425 1343 AEVRGDLAKNASGDALEIVAFNWNSKEKTTRRFPVSKIGRSHYRIVDFG 1391
Fsa16295Glu/1-454
Fsa11540/1-1154
Aga50D/1-793
AgaA/1-995
AgrA/1-995
PaBglu50A/1-749
VadG925/1-1425 1392 PVTLRDDLGVMLIPARNPAVRNLRIDRIILVPER 1425
Percent identity (%)
43
71
86

Supplementary Figure 1: Multiple sequence alignment of GH50 enzymes. MAFFT-DASH was used to generate a multiple sequence alignment of seven GH50s: Fsa16295Glu, Fsa11540, Aga50D, AgaA, AgrA, PaBglu50A, and VadG925. The alignment was visualized in Jalview v2.11.2.6. Two conserved glutamic acid residues of GH-A are highlighted in red, and the percent identity for each residue is indicated by purple shading.

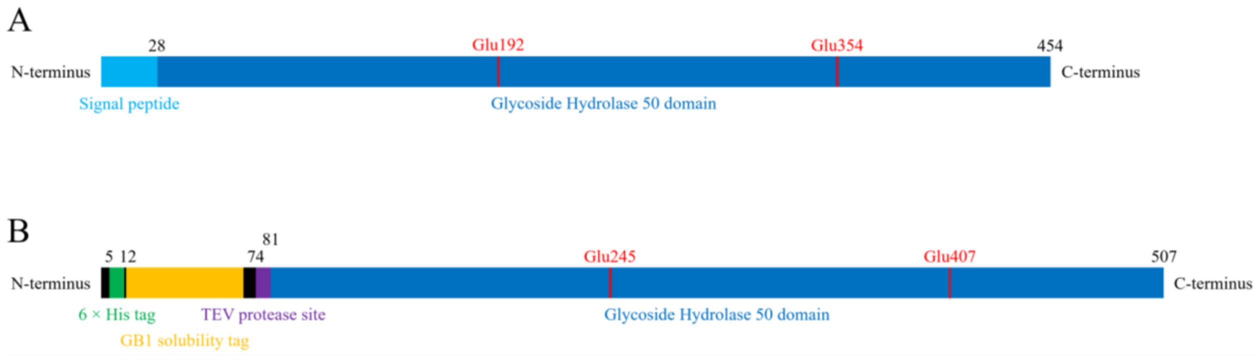

Supplementary Figure 2: Schematic of native and non-native Fsa16295Glu. (A) The native Fsa16295Glu protein containing an N-terminal signal peptide (light blue) and a glycoside hydrolase domain (blue) with conserved residues Glu192 and Glu354 (red). (B) The non-native Fsa16295Glu protein containing an N-terminal 6 × His tag (green), a GB1 solubility tag (yellow), a TEV protease site (purple), and short linkers between motifs (black), as well as a GH50 domain (blue) with conserved residues Glu245 and Glu407 (red).

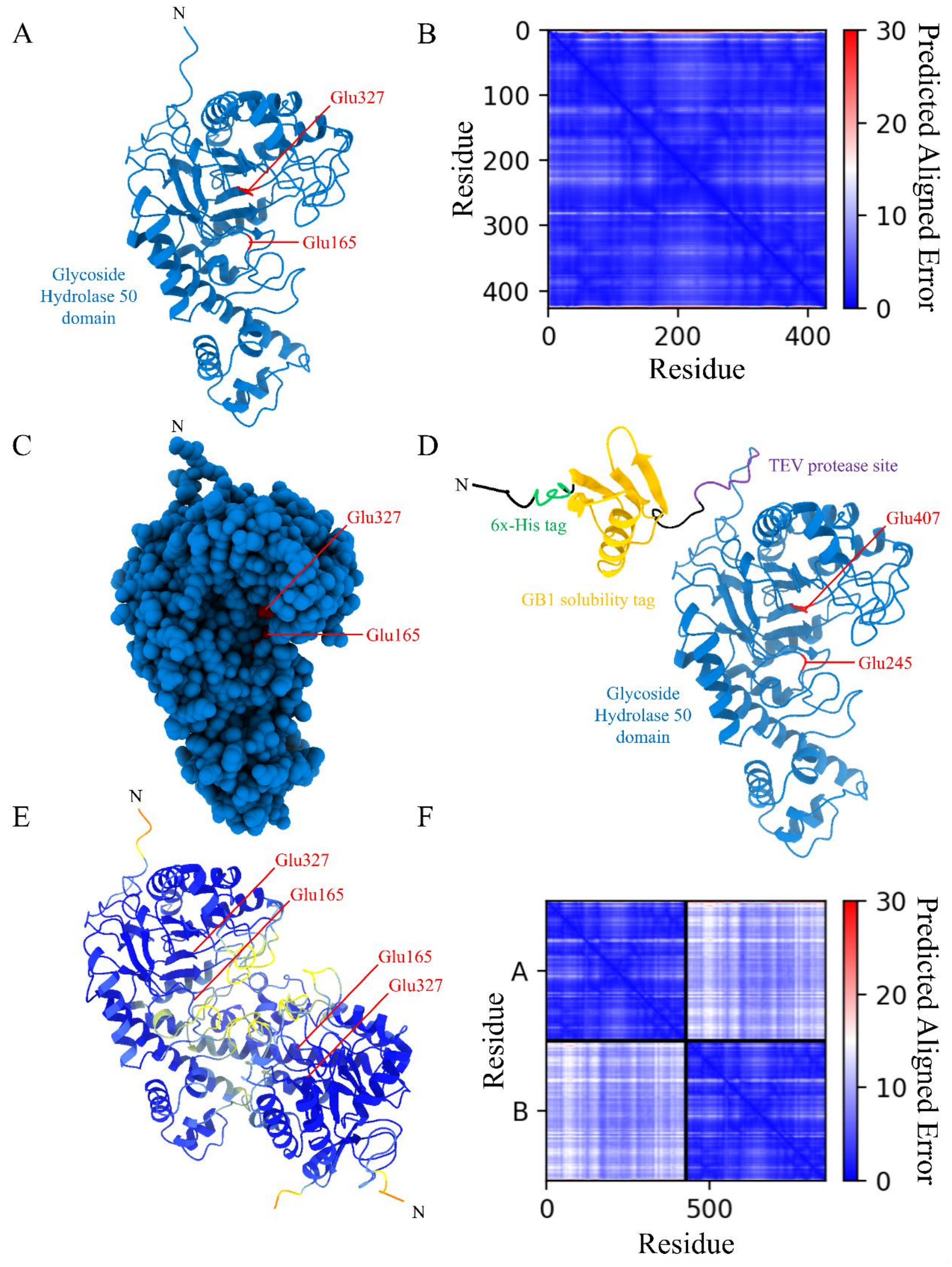
A
N
Glu327
Glu165
Glycoside
Hydrolase 50
domain
B
Predicted Aligned Error
Residue
Residue
C
N
Glu327
Glu165
D
N
6x-His tag
GB1 solubility tag
TEV protease site
Glu407
Glu245
Glycoside
Hydrolase 50
domain
E
N
Glu327
Glu165
Glu165
Glu327
N
F
Predicted Aligned Error
Residue
A
B
Residue

Supplementary Figure 3: AlphaFold 2 computed structure models of Fsa16295Glu. ColabFold integrated with ChimeraX v 1.5 was used to generate four Fsa16295Glu CSMs using AlphaFold 2. (A) CSMs of native, mature Fsa16295Glu (blue) with conserved residues Glu165 and Glu327 (red). (B) PAE plot of the native mature Fsa16295Glu CSM showing the distance error for each residue pair. (C) CSM surface representation of native, mature Fsa16295Glu (blue) with conserved residues Glu165 and Glu327 (red). (D) CSMs of non-native Fsa16295Glu containing an N-terminal 6 × His tag (green), GB1 solubility tag (yellow), and TEV protease site (purple), with conserved residues Glu245 and Glu407 (red). (E) Homodimeric CSM of native, mature Fsa16295Glu with predicted local distance difference test (pLDDT) coloring where residue confidence ranges from low (red) to high (blue). (F) PAE plot of the native, mature Fsa16295Glu homodimeric CSM revealing moderate confidence in the homodimer structure.

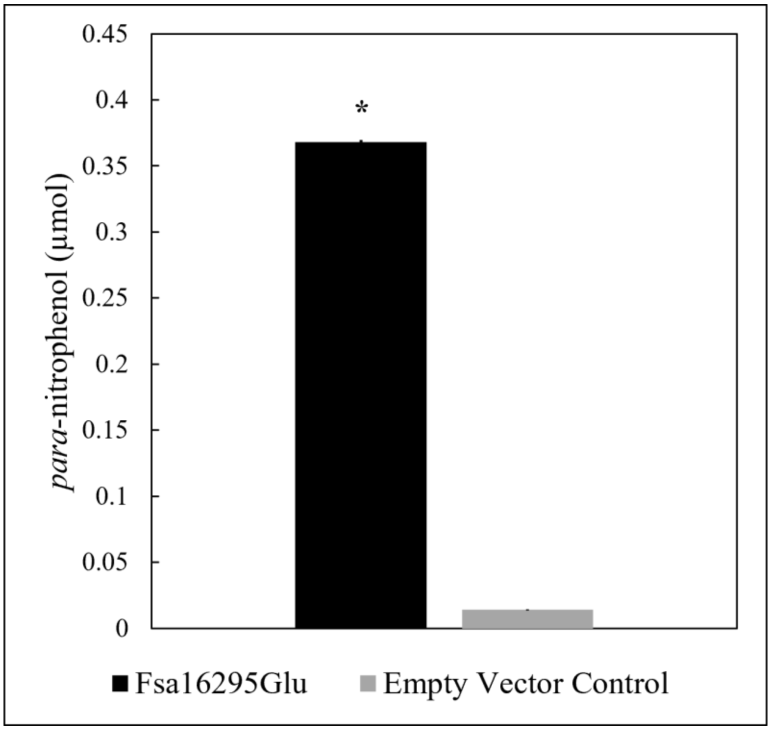

Supplementary Figure 4: *p*NPG activity assay. Fsa16295Glu is active on *p*NPG compared to an empty vector control (* $P \leq 0.000005$ via an unpaired t-test). Errors bars are based on standard deviation.

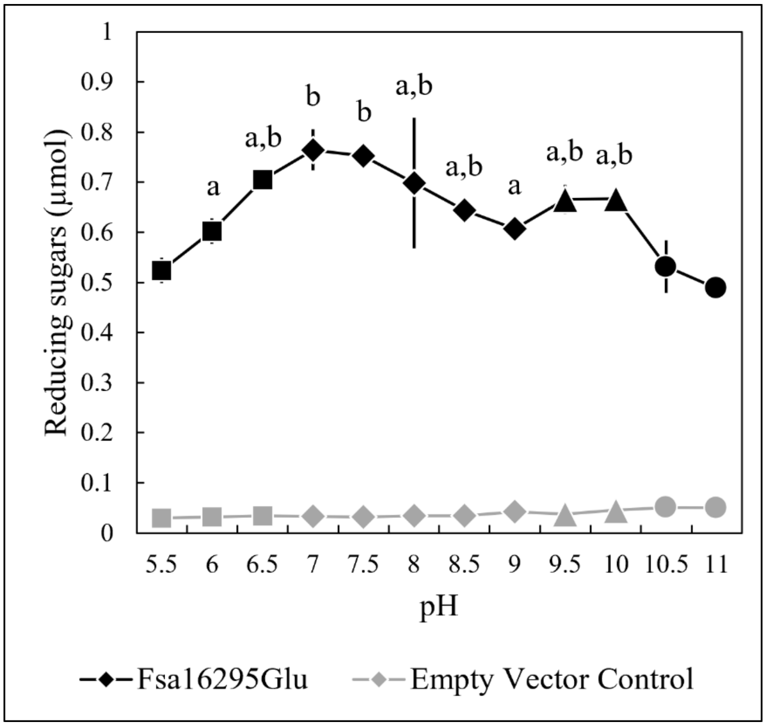

Supplementary Figure 5: pH range and optimum of Fsa16295Glu. Fsa16295Glu was active on oat β-glucan at all tested pH values, and optimally active between pH 6.5 and 10.0. pH values with a shared letter are not significantly different ($P \leq 0.05$ via a one-way ANOVA with post-hoc Tukey's HSD). The buffers used were MES (■, 5.5-6.5), Tris (♦, 7.0-9.0), CHES (▲, 9.5-10.0), and CAPS (●, 10.5-11.0). Error bars are based on standard deviation.

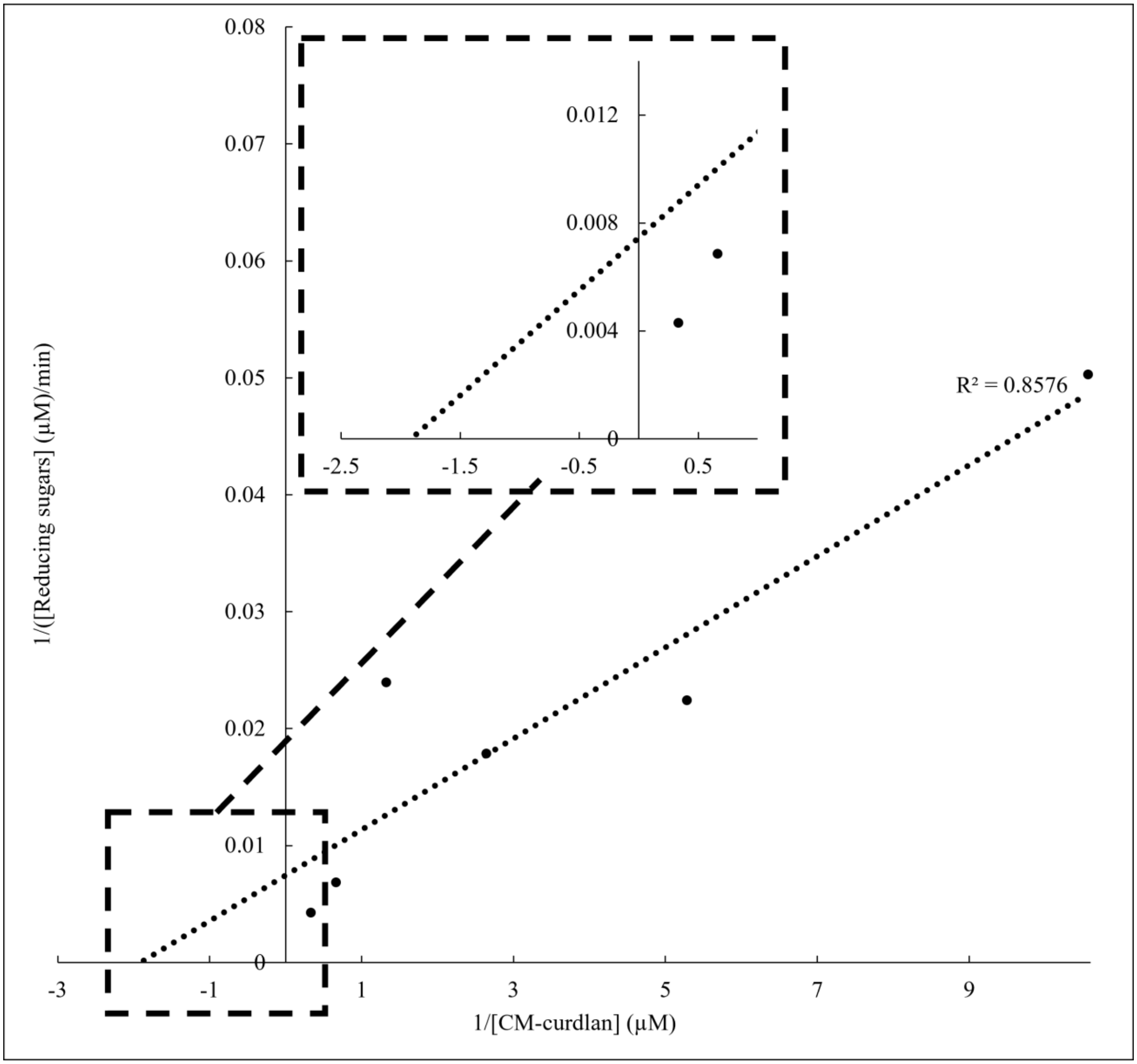

Supplementary Figure 6: Lineweaver-Burk plot for CM-curdlan. Incubation of Fsa16295Glu with varying concentrations of CM-curdlan for 30 minutes resulted in a linear Lineweaver-Burk plot ($R^2 = 0.8576$). The x- and y-intercepts were used to calculate the K_M and V_{max} values of 0.0520 µM and 133 µM/min, respectively. The x- and y-intercept region is magnified within a dashed box.

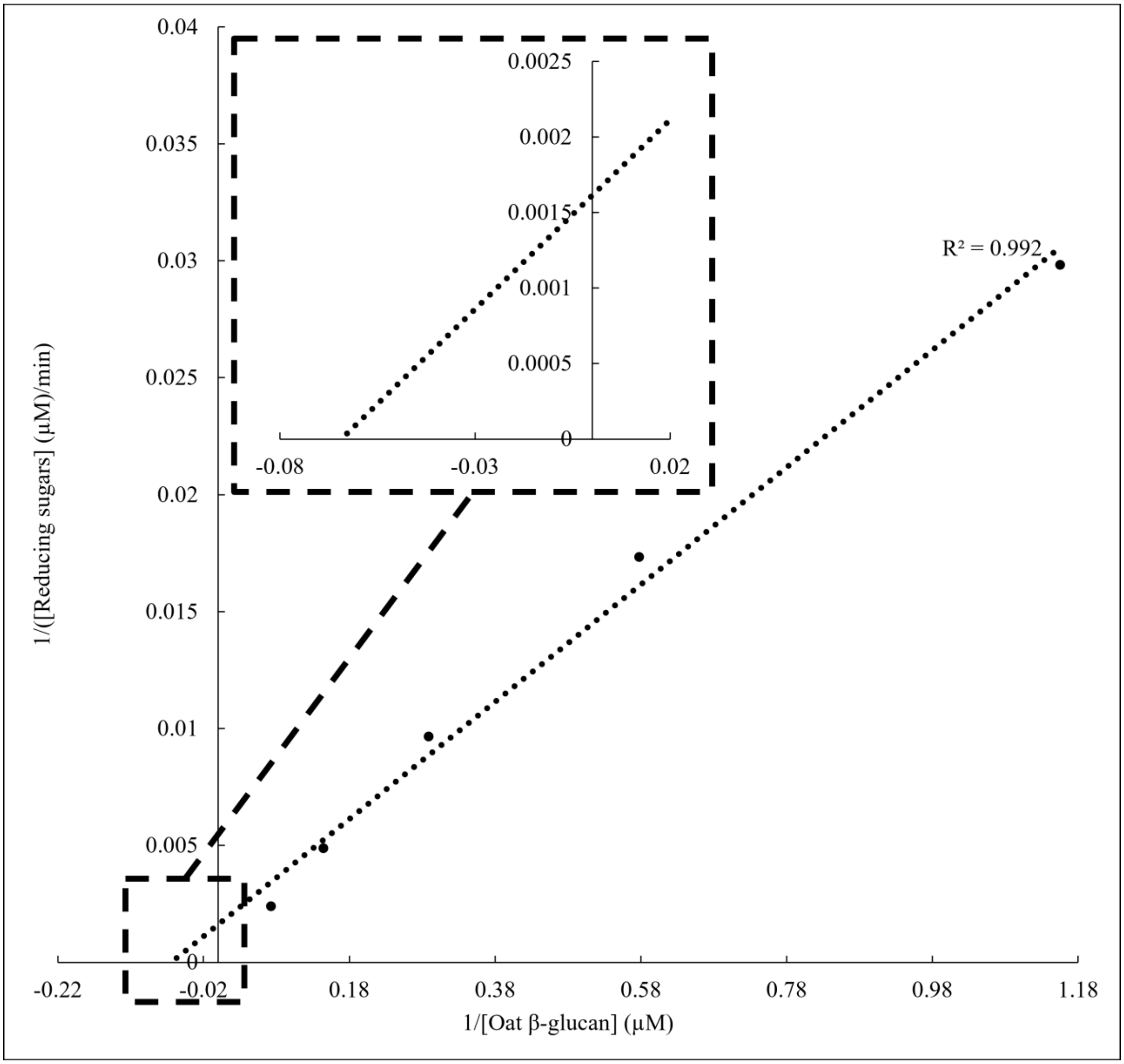

Supplementary Figure 7: Lineweaver-Burk plot for oat β-glucan. Incubation of Fsa16295Glu with varying concentrations of oat β-glucan for 30 minutes resulted in a linear Lineweaver-Burk plot (R^2 = 0.992). The x- and y-intercepts were used to calculate the K_M and V_{max} values of 15.7 μM and 625 μM/min, respectively. The x- and y-intercept region is magnified within a dashed box.

Supplementary Table 1: Characterized GH50 enzymes.

#	Enzyme name	GenBank accession number	Organism	Activity	Reference
1	VadG925	EFB02686.1	*Victivallis vadensis* ATCC BAA-548	β-galactosidase	https://doi.org/10.1007/s12275-012-2478-6
2	Ph1636	QEP52093.1	*Paraglaciecola hydrolytica* S66	β-agarase	https://doi.org/10.3389/fmicb.2018.00839
3	AgaB-4	AVV48109.1	*Paenibacillus agarexedens* BCRC 16000	β-agarase	https://doi.org/10.1186/s13568-018-0581-8
4	Hz1	ADY17918.1	*Agarivorans* sp. HZ105	β-agarase	https://doi.org/10.1111/j.1365-2672.2008.03990.x
5	agarase-B	BAG71427.1	*Vibrio* sp. PO-303	β-agarase	Araki et al., 1998
6	N/A	ABK97391.1	*Agarivorans* sp. JA-1	β-agarase	https://doi.org/10.1007/s10529-008-9634-4
7	AgaA	BAA03541.1	*Vibrio* sp. JT0107	β-agarase	https://doi.org/10.1128/aem.59.11.3750-3756.1993
8	AgrA	ACU52709.1	*Agarivorans* sp. AG17	β-agarase	https://doi.org/10.5657/FAS.2010.13.1.036
9	AgaA11	BAD99519.1	*Agarivorans* sp. JAMB-A11	β-agarase	https://doi.org/10.1042/BA20040083
10	Aga41A	ADM25828.1	*Vibrio* sp. CN41	β-agarase	https://doi.org/10.1128/AEM.05364-11
11	AgWH50A	AFP32918.1	*Agarivorans gilvus* WH0801	β-agarase	https://doi.org/10.1007/s11274-013-1591-y
12	Wh50B	AQT38174.1	*Agarivorans gilvus* WH0801	β-agarase	https://doi.org/10.1021/acs.jafc.7b01533
13	Hz2	ADY17919.1	*Agarivorans* sp. HZ105	β-agarase	https://doi.org/10.1111/jam.12389
14	AgaD02	ABM90422.1	*Agarivorans* sp. QM38	β-agarase	https://doi.org/10.1007/s13131-011-0098-3
15	AgaB	BAA04744.1	*Vibrio* sp. JT0107	β-agarase	https://doi.org/10.1128/aem.59.11.3750-3756.1993

16	Sco3487	CAB61811.1	*Streptomyces coelicolor* A32	β-agarase	https://doi.org/10.1128/JB.05978-11
17	AgWH50C	AHM94172.1	*Agarivorans gilvus* WH0801	β-agarase	https://doi.org/10.1007/s11274-013-1591-y
18	N/A	BAE97587.1	*Alteromonas* sp. E-1	β-agarase	https://doi.org/10.1016/s1389-1723(99)80091-7
19	Aga50D	ABD81904.1	*Saccharophagus degradans* 2-40	β-agarase	https://doi.org/10.1007/s00253-009-2256-5
20	Ph1609	QEP52089.1	*Paraglaciecola hydrolytica* S66	β-agarase	https://doi.org/10.3389/fmicb.2018.00839
21	AgaB1	AGT98631.1	*Thalassotalea agarivorans* BCRC 17492	β-agarase	https://doi.org/10.1111/jam.12389
22	Aga21	AHC72907.1	*Pseudoalteromonas* sp. NJ21	β-agarase	https://doi.org/10.1590/S1517-838246320131289
23	AgaA	ABD80438.1	*Saccharophagus degradans* 2-40	β-agarase	https://doi.org/10.1128/AEM.07004-11
24	Ph1624	QEP52091.1	*Paraglaciecola hydrolytica* S66	β-agarase	https://doi.org/10.3389/fmicb.2018.00839
25	PaBglu50A	AST24418.1	*Pseudomonas aeruginosa* CAU 342A	β-1,3-glucanase	https://doi.org/10.1016/j.enzmictec.2017.09.002
